# Material hardship, forced displacement, and negative health outcomes among unhoused people who use drugs in Los Angeles, California and Denver, Colorado: a latent class analysis

**DOI:** 10.1186/s12889-025-21626-6

**Published:** 2025-02-13

**Authors:** Jesse Lloyd Goldshear, Siddhi S. Ganesh, Annick Borquez, Lillian Gelberg, Karen F. Corsi, Ricky N. Bluthenthal

**Affiliations:** 1 Department of Medicine, Division of Infectious Diseases and Global Public Health, University of California San Diego School of Medicine, 9500 Gilman Drive #0507, La Jolla, CA 92093-0507 USA; 2 Department of Population and Public Health Sciences, Keck Medicine of University of Southern California, Los Angeles, USA; 3 Department of Family Medicine, David Geffen School of Medicine at UCLA, Los Angeles, USA; 4 Department of Psychiatry, University of Colorado School of Medicine, Aurora, USA; 5 Department of Health Policy and Management, Fielding School of Public Health, University of California Los Angeles, Los Angeles, USA

**Keywords:** Homelessness, People who use drugs, Latent class analysis, Drug overdose, Displacement

## Abstract

**Background:**

Homelessness is a growing concern in the United States, especially among people who use drugs (PWUD). The degree of material hardship among this population may be linked to worse health outcomes. PWUD experiencing homelessness in urban areas are increasingly subjected to policies and social treatment, such as forced displacement, which may worsen material hardship. It is critical to describe hardship among PWUD and examine if it is linked to health outcomes.

**Methods:**

Data were collected as part of a prospective cohort study of PWUD in Los Angeles, California and Denver, Colorado (*n* = 476). Analysis sample size was smaller (*N* = 395) after selecting for people experiencing homelessness and for whom data were complete. Five indicators assessing hardship (difficulty finding food, clothing, restrooms, places to wash/shower, and shelter) in the past three months were obtained from participants at baseline and were used in latent class analysis (LCA). We chose a base latent class model after examination of global fit statistics. We then built three auxiliary models using the three-step Bolck–Croon–Hagenaars (BCH) method to test the relationship of latent class membership to several hypothesized social and health variables in this same three month time period.

**Results:**

Fit statistics, minimum classification probabilities, and ease of interpretation indicated a three-class solution for level of material difficulty. We termed these classes “High Difficulty” (*n* = 82), “Mixed Difficulty” (*n* = 215), and “Low Difficulty” (*n* = 98). Average classification probabilities indicated good class separability. “High Difficulty” participants had high probabilities of usually having difficulty accessing all five resources. “Mixed Difficulty” participants indicated a range of difficulty accessing all resources, with restrooms and bathing facilities being the most difficult. “Low Difficulty” participants were defined by high probabilities of never having access difficulty. In auxiliary analyses, there were significant (*p* < 0.05) differences in experiences of displacement, opioid withdrawal symptoms, nonfatal overdose, and violent victimization between classes.

**Conclusions:**

This LCA indicates that among PWUD experiencing homelessness there exist distinct differences in resource access and material hardship, and that these differences are linked with political, social, substance use, and other health outcomes. We add to the literature on the relationship between poverty and health among PWUD. Policies which increase difficulty accessing necessary material resources may negatively impact health in this population.

**Supplementary Information:**

The online version contains supplementary material available at 10.1186/s12889-025-21626-6.

## Background

Unsheltered homelessness-defined by the United States Department of Housing and Urban Development as people whose “primary nighttime location is a public or private place not designated for, or ordinarily used as, a regular sleeping accommodation for people” —has been increasing yearly in the United States since 2015 [1]. As of 2023, national point-in-time estimates indicated nearly 230,000 people currently experiencing unsheltered homelessness on any given night, accounting for roughly 40% of the total homeless population in the US [2, 3]. Extant research attributes the causes of homelessness to a variety of structural and social factors including income inequality [4, 5], growing housing unaffordability [6], a lack of low-income housing in urban areas [7], overall rising costs for goods and services, and underinvestment in social safety net services and programs [8]. While a variety of individual-level factors have been found to contribute to pathways into homelessness, scholarship has shown that these individual-level factors may potentiate the impact of larger structural and social forces, rather than contribute directly on their own [9].

People experiencing homelessness, especially people who use drugs (PWUD), are at risk for a multitude of negative health outcomes as compared to their housed counterparts [10], including heightened risk of injury mortality [11], exacerbation of chronic health conditions, hepatitis B and C [12, 13], HIV outbreaks [14], and worsening mental health [15]. This population is also subject to various environmental exposures from air pollution [16], to lack of access to safe drinking water [17], extreme temperatures [18], and poor sanitary conditions [19]. Despite the reticence of many people experiencing homelessness to utilize medical care for fear of stigmatization and poor treatment [20, 21], unsheltered individuals are significantly more likely than those in shelters to utilize emergency department and outpatient services [22]. Unsheltered status has also been shown to increase risk factors for premature mortality [23].

### Material hardship

One contributing factor to these health risks is the level of *material hardship*-the inability to afford or access basic necessities of life [24, 25] -experienced by this community. Research across a variety of disciplines has documented the stark realities of contemporary urban homelessness. Unsheltered people have very little access to running water for drinking, bathing, or maintaining sanitation [26, 27]. Access to restrooms is often limited, with both public and private establishments often lacking convenient hours of operation or otherwise barring entry to people who ‘appear’ homeless [28, 29]. Nutritious food is difficult to find, and many people experiencing unsheltered homelessness subsist on a variety of donated or cheaply bought food that may not fulfill dietary guidelines or relieve hunger and may contribute to further exacerbation of chronic illness [30–33]. While ‘fast fashion’ has provided many in the Global North with access to relatively cheap clothing, people experiencing homelessness may not have either this same ability to purchase clothing nor the ability to adequately wash it when needed [34, 35]. These characteristics of material hardship go unassessed in single-item measures of socioeconomic status and income [36, 37]. A better understanding of the health impacts of material hardship among the most impoverished communities may aid policymakers and community-based organizations in prioritizing funding, materials, and services to where they are most needed.

Among this already socially marginalized and materially deprived population, (PWUD) may face both greater material hardship, and additional risks from encampment clearances and forced displacement policies like those recently declared constitutional in 2024 by the US Supreme Court [38]. While discrimination and stigma against unsheltered people is severe, PWUD are further marginalized by policies and practices that may bar them from key resources and increase their risk of experiencing drug-related harms, in addition to the above-mentioned negative health outcomes. Naloxone, a medication developed to reverse opioid overdose, available in multiple formulations and frequently distributed by harm reduction organizations, may be frequently lost during government-enforced displacement events [39–41]. Recently published research has also demonstrated an association between residential relocation of unsheltered PWUD and increased risk of nonfatal overdose and receptive syringe sharing, higher odds of recent arrest and being jailed, and a predicted rise in mortality [42, 43].

Links between access to material resources and both experiences of violence and non-fatal overdose have been established across multiple study populations. Based on this prior research on material deprivation and access to resources among populations of PWUD, we expected a relationship between difficulty accessing material resources and experience of displacement, non-fatal overdose [44, 45], experiences of withdrawal, and experience of physical violence [46–48].

### Hypotheses

In response to increases in unsheltered homelessness, growing criminalization of unsheltered PWUD, and deepening material uncertainty among this population, we conducted an exploratory latent class analysis (LCA) and follow-up auxiliary analyses to characterize profiles of material hardship in this community and their association with health outcomes. LCA is a method to group subjects from multivariate data into ‘latent classes’-subgroups with different qualities [49]. This can enable researchers to identify distinct unobserved risk profiles, behavioral patterns, or other characteristic groupings based on a combination of observed variables. We used five questionnaire items measuring difficulty in accessing essential resources (“competing needs”) as originally utilized by Gelberg et al. [50] with permission from the author. We tested the following hypotheses in this analysis:

- H1) In LCA at least a two-class solution would emerge that indicated high and low levels of past 3-month material hardship,
- H2) in auxiliary models the class membership would be significantly associated with probability of 2a) past three-month displacement, 2b) any past three-month opioid withdrawal symptoms, 2c) past three-month nonfatal overdose experience, and 2d) past three-month violent victimization.

## Methods

### Participants

This study uses data collected as part of a prospective cohort study of people who inject drugs (PWID) (*N* = 476) in Los Angeles, California and Denver, Colorado from April 2020 to February 2023. Baseline data used here were collected primarily during 2020 and 2021, representing peak years of the COVID-19 pandemic. Participants were recruited as part of a convenience sample from community-based sites (syringe services programs, homeless service organizations), and were administered computer-assisted questionnaires by research team staff [51]. Eligible participants were 1) 18 years of age or older, 2) could converse in and read English fluently, 3) reported injection of any illicit drug in the prior month, and 4) reported use of any illicit opioid in the prior month. Administered questionnaires lasted between 30 min and one hour.

Questionnaire domains relevant to this analysis included *demographics, substance use patterns and practices, housing and other living accommodations, and violent victimization*. Participants were compensated $20 for their time. While most of the study population was currently experiencing homelessness at baseline, a minority (*n* = 77, 16.3%) were housed at the time of data collection. For this analysis we used only the subset of participants who were experiencing homelessness (*N* = 395) in the past three months. This protocol was approved by the Institutional Review Boards of the University of Southern California.

### Variables

Cross-sectional baseline questionnaire data were utilized for all components of this analysis. Five variables were used as indicator items in construction of the base latent class model for material difficulty: difficulty of finding food, clothing, a place to shower/wash, a place to use the bathroom, and shelter in the past three months. These items were rated on an ordinal four-point scale of Usually (4), Sometimes (3), Rarely (2), or Never (1). Demographic independent variables included city of residence (binary), age (continuous), gender identity (categorical), race/ethnicity (categorical), past-year monthly income (categorical), and years of continuous homelessness at time of recruitment (categorical). Dependent variables of interest for hypothesis testing were past three-month experiences of displacement (categorical), past three-month opioid withdrawal symptoms (binary), past three-month nonfatal overdose (binary), and past three-month violent victimization (binary, defined as either having been threatened with a weapon or being physically assaulted with or without a weapon).

### Latent class analysis

In the following analysis, we followed the steps outlined by Van Lissa, Garnier-Villarreal, and Anadria in their SMART-LCA checklist for conducting exploratory LCA with ordinal indicators [52]. After examining the distribution of our indicator items we checked for missingness on all variables. No missingness was observed. To obtain a base latent class model (H1), we iteratively constructed models ranging from one to five latent classes. We stopped at a five-class solution as the number of classes cannot exceed the number of model items. We selected a final base model solution using global fit statistics including likelihood ratio tests, information criteria (Aikake information criterion [AIC], Bayesian information criterion [BIC], and sample-size adjusted Bayesian information criterion [saBIC]), and entropy. Among model solutions where these test results were contradictory, we removed models from contention based on local identifiability and the theoretical interpretation of results [49, 52–54].

### Auxiliary model fitting for demographics and hypothesis testing

After we identified the base model we used a three-step procedure developed by Bolck, Croon, and Hagenaar (BCH) [55] as implemented in Van Lissa, Garnier-Villarreal, & Anadria [52] to assess probable demographic composition of each class and to test our stated hypotheses in bivariate auxiliary models (H2a, H2b, H2c, and H2d). We examined the reported omnibus likelihood ratio tests and associated *p*-values to determine the significance of auxiliary variable differences across and between classes. All analyses were conducted in Base R [56] using R Studio [57], and primarily utilized the tidySEM [58] and ggplot2 [59] packages.

## Results

### Variable comparisons by city: demographics, material difficulty, and outcomes

Participant age ranged from 19 to 76 years, with a mean of 39.8 (standard deviation [SD] = 10.5). The majority of the sample was male (76.4%) and White (51.9%). The largest non-White minority groups in the sample were Latino/a (26.0%) and Indigenous/Native American (10.6%). While monthly income in this participant population varied, the majority (52.4%) reported earning less than $1,000 USD per month. The majority of the sample indicated a period of homelessness of less than five years (68.6%). The study population had the most material difficulty in the past 3 months finding both restrooms and a place to bathe, and the least difficulty obtaining clothing. Experiences of government enforced displacement (62%), opioid withdrawal symptoms (76.5%), nonfatal overdose (24.1%), and violent victimization (47.1%) were all common. Participant age and race/ethnicity differed significantly (*p* < 0.05) by participant location, as did difficulty finding clothing, restrooms, places to bathe, and displacement experiences. Participants in Denver were significantly younger, whiter, had greater material difficulties, and were more-often forcibly displaced. All comparisons can be found in Table 1.

**Table 1:** Participant demographic, material hardship, and outcome variable characteristics

Characteristic	Mean (SD) or *N* (%)			
**Demographics**	Los Angeles (*n* = 167)	Denver (*n* = 228)	Total (*N* = 395)	*p*
**Age**	41.2 (11.6)	38.7 (9.5)	39.8 (10.5)	0.02
**Gender**				
Male	128 (76.6)	184 (80.7)	312 (79.0)	0.61
Female	35 (21.0)	40 (17.5)	75 (19.0)	
Other	4 (2.4)	4 (1.8)	8 (2.0)	
**Race/Ethnicity**				
White	76 (45.5)	136 (59.6)	212 (53.7)	< 0.001
Black	14 (8.4)	7 (3.1)	21 (5.3)	
Latinx	57 (34.1)	37 (16.2)	94 (23.8)	
Indigenous	6 (3.6)	37 (16.2)	43 (10.9)	
Other	14 (8.4)	11 (4.8)	25 (5.3)	
**Monthly Income**^**a**^				
< $1,000	87 (52.7)	126 (55.8)	213 (53.9)	0.77
$1,000 > $1,400	32 (19.4)	37 (16.4)	69 (17.5)	
$1,401 > $2,100	21 (12.7)	33 (14.6)	54 (13.7)	
> $2,101	25 (15.2)	30 (13.3)	55 (31.9)	
**Length of Homelessness**^**b**^				
< 1 Year	53 (31.9)	71 (31.1)	124 (31.4)	0.19
1 to 5 years	53 (31.9)	94 (41.2)	147 (37.2)	
5 to 10 years	38 (22.3)	43 (18.9)	81 (20.5)	
> 10 years	22 (13.3)	20 (8.8)	42 (10.6)	
**Material Hardship**^**c**^				
**Clothing**				
Never	80 (47.9)	79 (34.6)	159 (40.3)	0.02
Rarely	11 (6.6)	25 (11.0)	36 (9.1)	
Sometimes	40 (24.0)	53 (23.2)	93 (23.5)	
Usually	36 (21.6)	71 (31.1)	107 (27.1)	
** Food**				
Never	73 (43.7)	70 (30.7)	143 (36.2)	0.05
Rarely	17 (10.2)	36 (15.8)	53 (13.4)	
Sometimes	44 (26.3)	68 (29.8)	112 (28.4)	
Usually	33 (19.8)	54 (23.7)	87 (22.0)	
** Restrooms**				
Never	57 (34.1)	41 (18.0)	98 (24.8)	< 0.001
Rarely	10 (6.0)	16 (7.0)	26 (6.6)	
Sometimes	45 (26.9)	55 (24.1)	100 (25.3)	
Usually	55 (32.9)	116 (50.9)	171 (43.3)	
** Shelter**				
Never	63 (37.7)	67 (29.4)	130 (32.9)	0.24
Rarely	17 (10.2)	31 (13.6)	48 (12.2)	
Sometimes	32 (19.2)	56 (24.6)	88 (22.2)	
Usually	55 (32.9)	74 (32.5)	129 (32.7)	
** Shower/Bath**				
Never	60 (35.9)	48 (21.1)	108 (27.3)	0.004
Rarely	12 (7.2)	21 (9.2)	33 (8.4)	
Sometimes	42 (25.1)	54 (32.7)	96 (24.3)	
Usually	53 (31.7)	105 (46.1)	158 (40.0)	
**Outcomes**^**c**^				
**Displacement**				
Not Displaced	65 (38.9)	34 (14.9)	99 (25.1)	< 0.001
Voluntarily Moved	29 (17.4)	22 (9.6)	51 (12.9)	
Displaced By Government	73 (43.7)	172 (75.4)	225 (62.0)	
**Opioid Withdrawal Symptoms**	122 (73.1)	180 (78.9)	302 (76.5)	0.2136
**Nonfatal Overdose**	41 (24.7)	54 (23.7)	95 (24.1)	0.9099
**Violent Victimization**	72 (43.1)	114 (50.0)	186 (47.1)	0.2104

^a^4 observations missing data on monthly income

^b^1 observation missing data on homelessness length

^c^Past three months

### Base model fit and selection

After comparison of fit indices and examination of potential class interpretability, we selected the three-class model (H1) for level of material difficulty PEH experienced in the past three months. Information criteria gave conflicting results (Table 2). The three-class solution had the lowest BIC, the four-class solution contained the lowest saBIC, while the five-class solution yielded the lowest AIC. Given these results, we moved on to comparisons of entropy and parameter-observation ratios to inform model selection. Minimum classification probability decreased with each additional class solution indicating increasingly poor class distinction. Global and local ratios of observations to model parameters also continued decreasing and indicated very few observations in the smallest classes of the four- and five-class solutions. Thus, we eliminated the four- and five-class models despite the five-class solution providing the best overall entropy value (excluding the one-class solution) and selected the three-class solution as our base model. We did however attempt to interpret all potential class solutions regardless of fit in case those we removed from contention yielded theoretically valuable information. The poor class distinctions of the larger models made them difficult to interpret or justify theoretically and reaffirmed our choice of base model.

**Table 2:** Fit and information criteria for one through five class models for level of material hardship

**Classes**	LL*	**I**	**Parameters**	AIC**	BIC***	**saBIC**^†^	**Entropy**	**Minimum Classification Probability**	**Proportion of Population in Smallest Class**	**Observations per Parameter**	**Observations per Parameter in Smallest Class**
1	-2,542.28	395	15	5,114	5,174	5,126	1.00	1.00	1.00	26.33	26.33
2	-2,344.37	395	31	4,750	4,874	4,775	0.76	0.89	0.37	12.74	9.87
3	-2,268.97	395	47	4,631	4,818	4,669	0.81	0.88	0.21	8.40	5.47
4	-2,228.61	395	63	4,583	4,833	4,633	0.75	0.81	0.17	6.27	4.60
5	-2,209.42	395	79	4,576	4,891	4,640	0.83	0.76	0.12	5.00	3.20

^*^Log Likelihood

^**^Akaike Information Criterion

^***^Bayesian Information Criterion

^†^Sample size adjusted Bayesian Information Criterion

### Base model interpretation

**Fig. 1:**
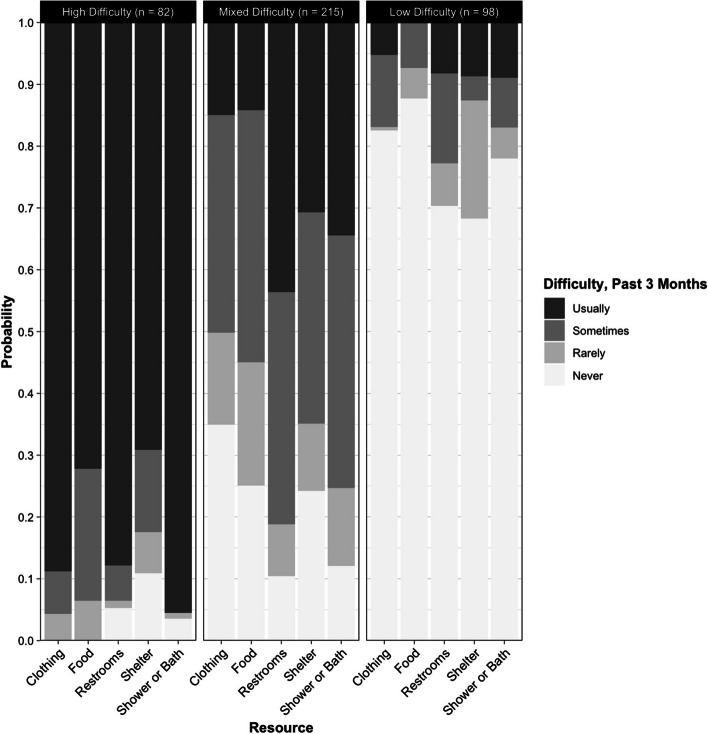
Item response probabilities by Class and Resource

Average classification probabilities (Supplemental Table 1) indicate good class separation and identification (Fig. 1) for level of material difficulty. Mean assignment probability was 89.1% for class 1 “High Hardship” participants (*n* = 82), 91.8% for class 2 “Mixed Hardship” participants (*n* = 215), and 91.1% for class 3 “Low Hardship” participants (*n* = 98). Those participants most likely assigned to the “High Hardship” class were characterized by greater than 65% probability of endorsing usual past 3-month difficulty finding all five resources, with the highest probability (95.5%) of usual difficulty finding a place to bathe and lowest probability (69.1%) of usually accessing shelter. For participants most likely assigned to the “Mixed Hardship” class, none of the difficulty ranks for any resource were endorsed with less than 11% probability or more than 44% probability. They indicated the greatest difficulty finding restrooms (usually = 43.7%, sometimes = 37.5%) and a place to bathe (usually = 40.8%, sometimes = 34.5%), and the least difficulty finding clothing (never = 34.9%, rarely = 14.9%). Participants assigned to the “Low Hardship” class were characterized by a greater than 65% probability of endorsing never having past 3-month difficulty accessing any of the five resource categories, with food being the easiest to access resource (never = 87.7%, rarely = 4.9%), and restrooms being the most difficult (usually = 8.2%, sometimes = 14.6%).

### Demographic auxiliary models

We found significant differences in recruitment location (*p* = 0.0004), participant age (*p* = 0.0043), and participant gender (*p* = 0.0186) between latent classes of material hardship (Table 3). Class membership probability was not significantly different for participant race/ethnicity, monthly income, or years homeless. Denver-based participants had the highest probability of membership in the “high hardship” class, while Los Angeles-based participants were most probably assigned to the “low hardship” class. Mean age was highest among “low hardship” participants. While the “high hardship” and “low difficulty” classes were not significantly different regarding participant gender, males were more probably assigned to the “mixed hardship” class.

**Table 3:** BCH^a^-Estimated Demographic Comparisons By Class Assignment

Demographic Characteristic	High vs. Mixed		High vs. Low		Mixed vs. Low		Overall	
	LL^b^ Difference	*p*	LL Difference	*p*	LL Difference	*p*	LL Difference	*p*
Recruitment Location	4.2426	0.0394	15.0694	0.0001	6.8408	0.0089	15.4857	0.0004
Age	2.8617	0.2391	13.5831	0.0011	8.2065	0.0165	15.2151	0.0043
Race	4.3420	0.3617	1.0683	0.8993	7.5532	0.1094	9.6138	0.2932
Gender	6.5253	0.0383	0.4920	0.7819	8.9179	0.0116	11.8380	0.0186
Monthly Income	2.3647	0.5002	3.2431	0.3556	2.4080	0.3556	5.1550	0.5241
Years Homeless	1.0590	0.7870	1.2573	0.7393	0.9089	0.8233	2.0789	0.9123
	**Class 1: High**		**Class 2: Mixed**		**Class 3: Low**			
	P^c^(Membership) or Mean		P(Membership) or Mean		P(Membership) or Mean			
**Recruitment Location**								
Los Angeles	0.28		0.41		0.57			
Denver	0.72		0.59		0.43			
**Age**	37.56		39.25		42.63			
**Race**								
White	0.50		0.59		0.46			
Black	0.03		0.06		0.06			
Latinx	0.26		0.21		0.28			
Native American/Indigenous	0.14		0.08		0.15			
Other	0.06		0.07		0.05			
**Gender**								
Male	0.72		0.85		0.72			
Female	0.26		0.15		0.23			
Other	0.03		0.01		0.04			
**Monthly Income**								
< $1,000	0.61		0.53		0.51			
$1,001—$1,500	0.16		0.16		0.22			
$1,501—$2,000	0.13		0.15		0.11			
> $2,000	0.09		0.15		0.15			
**Years Homeless**								
< One Year	0.33		0.30		0.33			
One to Five Years	0.38		0.39		0.34			
Five to Ten Years	0.33		0.21		0.23			
> Ten Years	0.12		0.10		0.10			

^a^Bolck, Croon, and Hagenaars three-step method

^b^Log-Likelihood

^c^Probability

### Auxiliary models testing hypotheses

In testing hypotheses 2a, 2b, 2c, and 2d we found statistically significant differences in auxiliary variable probability by most likely material hardship class membership (Table 4). In all four bivariate auxiliary models, there was no significant difference between the “high hardship” and “mixed hardship” classes. Both “high hardship” and “mixed hardship” participants had higher probability of experiencing past 3-month government enforced displacement (Log likelihood difference [∆LL] = 24.4 [*p* < 0.001] and ∆LL = 22.0 [*p* < 0.001] respectively), opioid withdrawal symptoms (∆LL = 7.0 [*p* = 0.008] and ∆LL = 5.6 [*p* = 0.019] respectively), nonfatal overdose (∆LL = 6.7 [*p* = 0.010] and ∆LL = 16.5 [*p* < 0.001] respectively), and violent victimization (∆LL = 12.4 [*p* < 0.001] and ∆LL = 9.3 [*p* = 0.002] respectively) than did “low hardship” participants.

**Table 4:** BCH^a^-Estimated Health Risk Comparisons By Class Assignment

Health Risk	High vs. Mixed		High vs. Low		Mixed vs. Low		Overall	
	LL^b^ Difference	*p*	LL Difference	*p*	LL Difference	*p*	LL Difference	*p*
Forced Displacement	3.3190	0.1902	24.3518	0.0000	21.9534	0.0000	31.4017	0.0000
Opioid Withdrawal Symptoms	0.8173	0.3660	6.9985	0.0082	5.5521	0.0185	8.3670	0.0152
Nonfatal Overdose	0.7495	0.3845	6.6736	0.0098	16.4504	0.0000	16.4970	0.0003
Violent Victimization	1.5009	0.2205	12.3851	0.0004	9.3154	0.0023	14.1561	0.0008
	**Class 1: High**		**Class 2: Mixed**		**Class 3: Low**			
	P^c^ (Membership)		P(Membership)		P(Membership)			
Forced Displacement								
Not Displaced	0.12		0.21		0.43			
Moved Voluntarily	0.14		0.11		0.17			
Displaced By Government	0.74		0.68		0.40			
	P(No)	P(Yes)	P(No)	P(Yes)	P(No)	P(Yes)		
Opioid Withdrawal Symptoms	0.17	0.83	0.21	0.79	0.34	0.66		
Nonfatal Overdose	0.75	0.25	0.70	0.30	0.90	0.10		
Violent Victimization	0.42	0.58	0.50	0.50	0.68	0.32		

^a^Bolck, Croon, and Hagenaars three-step method

^b^Log-Likelihood

^c^Probability

## Discussion

In this exploratory latent class analysis, we showed that a sample of PWID experiencing homelessness could be categorized into multiple classes of material hardship and that class membership was associated with multiple social and health outcomes including forced displacement, experiences of opioid withdrawal symptoms, nonfatal overdose, and violent victimization. While novel, these results are firmly situated within the extant literature on material deprivation and resource access among people who use drugs. A New York City study that comprehensively captured [60] poor material conditions among PWUD via their 18-item adapted scale that included other aspects of resource deprivation, including transport, phone, medical access, money and income, and time for sleep also reported wide variation in socioeconomic marginalization and material deprivation similar to our study. Studies in other populations of PWUD have utilized their measure in relation to health outcomes-including two tested in this study-such as adherence to antiretroviral therapy for HIV [61], street-based violence [48], and non-fatal overdose [45]. Like van Draanen et al., (2021) and Mitra (2022), our extracted latent classes of resource access difficulty showed significant associations between higher material hardship and both non-fatal overdose and exposure to violence. Of note, even among a population comprised solely of people experiencing homelessness who use opioids, of which one might expect near universal experiences of withdrawal symptoms in a fentanyl-saturated market [62], our analysis indicates that participants experiencing the least material hardship had a significantly lower probability of experiencing recent opioid withdrawal. Those participants experiencing less material hardship may have had overall more-stable lives (which comports with the authors’ prior qualitative work on material and psychological security [63]) and thus may have had more consistent access to drugs, or access to a more consistent drug market.

Our results are further contextualized within the broad scope of quantitative and qualitative academic work linking poverty and health outcomes among both people who use and people who do not use drugs. These outcomes-increased risk of bloodborne pathogen infection, increased risk and severity of skin and soft tissue infection, exacerbation of chronic physical and mental health conditions—are all directly and indirectly linked to socioeconomic status and marginalization [64, 65]. Indirectly, impoverishment and deprivation are linked to health outcomes among PWUD by impacting multiple risk behaviors, including changes in substance use behaviors and income generation strategies [66–68]. Further effects of resource deprivation on health can be observed in the relationship between poverty and access to health resources, exposure to environmental hazards, and social isolation and abandonment [69–71]. These changes in behavior and production of health risk are modified by intersectional characteristics like age, race/ethnicity, and gender [72, 73] —some of which we also observed to differ significantly between latent classes in our model. While we did not test the impact of these intersectional characteristics, we demonstrated that the relationship between resource access difficulty and health outcomes is true even among PWUD who are already experiencing homelessness. Restrooms and bathing facilities were most difficult to access across extracted classes of material hardship, indicating a broad need for investment in availability of these services. While we did not include other material needs such as income or access to healthcare, this analysis also serves as a test case for Whittle et al.’s (2020) [74] precarity and health framework, wherein they theorized the ways in which co-occurring material-need insecurities result in poor health outcomes. Our analysis revealed that our participant population of unhoused PWUD was not homogenous in their experience of some aspects of material-needs precarity. With the addition of additional indicator items in a larger population sample, precarity and hardship and their relationship to health outcomes could be more fully tested.

Importantly, our analysis considered outcomes beyond the traditional health realm in that we include displacement and violent victimization as health outcomes, showing that experiences of material hardship are more severe among those exposed to forced displacement and victimization. The impact of forced displacement on loss of personal belongings, including cash, clothing, and medications, has been characterized in several studies [41, 63, 75], supporting one interpretation of the relationship observed in our analysis. We expect that forced displacement exacerbates situations of already-extreme vulnerability, leading to poor material difficulty and health outcomes. However, a situation of even moderate material hardship also likely increases the odds of being forcefully displaced, as it reflects more chronic, desperate, and publicly visible unsheltered conditions. This public visibility increases the likelihood that authorities and housed community members will call for displacement. The observed relationship between material hardship and violent victimization is likely also bidirectional. Multiple measures of poverty and insecurity have been linked to violent victimization [76], and our results show that this effect may manifest on a smaller scale even among an already highly marginalized, housing-insecure population. However, chronic exposure to violence likely inflicts trauma that makes attaining wellbeing and access to material resources less likely. These outcomes highlight the need for integrated approaches addressing both trauma and access to basic needs.

While fit statistics and class distinction guided us towards the three-class model, in further auxiliary testing, there was no observed significant difference in these outcomes between the”high” and “mixed” classes. There are several methodological explanations for this, including the analytic limitations listed below. It is also possible that despite the statistical distinction between these classes, health outcomes all become equally likely beyond a certain threshold of material hardship. Although this potential explanation seems to disagree with the literature, we feel that it justifies the need for further research with larger sample sizes and more robust data collection. Recruitment location was the only demographic variable for which class assignment probability differed significantly across all three models. While this may be due to differences in resource availability between the two cities, we feel it is likely an artifact of recruitment location within Los Angeles. One of our recruitment sites was in an area of highly concentrated service availability (Skid Row). While the population here is highly impoverished and unhoused, there are is also a high density of material resources. This is likely to have driven the difference in material resource access between Los Angeles and Denver.

This analysis has several limitations. Data were collected from a population of PWUD in locations that are currently experiencing a growth in visible unsheltered homelessness and overall changes in drug use against a backdrop of punitive and carceral homelessness policies including forced displacement. While we feel this makes the results relevant to the current moment, it may also limit their overall generalizability, and as such, the results of this study may not necessarily be applicable to other locations whose demographics, drug markets, and policies do not mirror that of the study locations. Data were also collected during the ongoing COVID-19 pandemic, which may have influenced both participant recruitment and resource availability. Results presented are also cross-sectional and resulting associations cannot be interpreted as causal—something that future longitudinal research could elucidate. In addition to being cross-sectional, auxiliary models represent bivariate, unadjusted differences in outcome probability between latent classes, and as such are likely subject to confounding that we were unable to measure as part of this analysis. While we attempted to describe difficulty of access to ‘bare necessities’ (food, clothing, shelter, hygiene) with these measures, by design there are aspects of material hardship and economic deprivation that our latent classes cannot capture, such as relational and psychological domains, and access to other material needs like phones [77, 78]. Given the limits of our data collection, we were also unable to assess any relationship between hardship and fatal overdose, which may be different than the relationship with nonfatal overdose. Finally, as with all self-report survey data, our results may be biased by participant recall and social desirability.

## Conclusions

We feel this analysis captures intuitive but important material dimensions of impoverishment and social marginalization that are often subsumed into single-item measures of socioeconomic status and income [36, 37] and can help towards the prioritization of harm reduction and welfare interventions both in terms of the groups to be reached and the most immediate needs that should be addressed. Monthly income was not significantly associated with differences in material hardship class membership probability, indicating that the material hardship of resource access among this population is unrelated to reported income—a traditional measure of impoverishment. Our study adds to the extant literature by showing that among already-homeless PWUD, resource access is still an important dimension of poverty and could be linked to multiple substance use and other health outcomes including withdrawing from opioids, experiencing nonfatal opioid overdose, and becoming a victim of violence. Additionally, given the ongoing political backlash to highly visible unsheltered houselessness, we feel that it is of importance to demonstrate the potential outcomes of policies, such as forced displacement, that lead to further marginalization for PWUD experiencing homelessness [43, 63]. Further research is needed, however, to fully explore the direct and indirect impacts of resource deprivation on downstream health outcomes. This includes both more comprehensive causal analysis and qualitative analysis that can report on and contextualize the experiences of unsheltered communities.

## Supplementary Information

Supplementary Material 1.

Supplementary Material 2.

## Data Availability

The datasets generated and/or analyzed during the current study are not publicly available due to contents which could potentially identify research participants engaged in illegal activity, but are available from the corresponding author [JLG] and last author [RNB] on reasonable request.

## Abbreviations

PWUD: People who use drugs
LCA: Latent class analysis
BMC: Bolck-Croon-Haanegar
PWID: People who inject drugs
AIC: Aikake information criterion
BIC: Bayesian information criterion
SaBIC: Sample-size adjusted Bayesian information criterion
∆LL: Log-likehood difference
