# Supplementary material for: Material hardship, forced displacement, and negative health outcomes among unhoused people who use drugs in Los Angeles, California and Denver, Colorado: a latent class analysis
Source: BMC Public Health. 2025 Feb 13;25:591. doi: 10.1186/s12889-025-21626-6 (PMC11823192; doi:10.1186/s12889-025-21626-6)
Supplement: Supplementary file 1 — Supplementary Material 1. [file 12889_2025_21626_MOESM1_ESM.docx]

| **Supplemental Table 1: Item Response and Classification Probabilities for Three-Class Model for Level of Material Hardship** | | | | |
| --- | --- | --- | --- | --- |
| Item Response Probabilities by Class | | **1 (n = 82)** | **2 (n = 215)** | **3 (n = 98)** |
| *Type of Difficulty* | *Frequency of Difficulty* |  |  |  |
| Clothing | *Never* | 0.0000 | 0.3492 | 0.8256 |
| Clothing | *Rarely* | 0.0432 | 0.1490 | 0.0055 |
| Clothing | *Sometimes* | 0.0692 | 0.3520 | 0.1167 |
| Clothing | *Usually* | 0.8876 | 0.1498 | 0.0522 |
| Food | *Never* | 0.0000 | 0.2504 | 0.8773 |
| Food | *Rarely* | 0.0645 | 0.1999 | 0.0488 |
| Food | *Sometimes* | 0.2137 | 0.4080 | 0.0739 |
| Food | *Usually* | 0.7219 | 0.1418 | 0.0000 |
| Restrooms | *Never* | 0.0525 | 0.1044 | 0.7031 |
| Restrooms | *Rarely* | 0.0120 | 0.0839 | 0.0691 |
| Restrooms | *Sometimes* | 0.0568 | 0.3753 | 0.1459 |
| Restrooms | *Usually* | 0.8786 | 0.4365 | 0.0820 |
| Shelter | *Never* | 0.1089 | 0.2421 | 0.6831 |
| Shelter | *Rarely* | 0.0664 | 0.1088 | 0.1908 |
| Shelter | *Sometimes* | 0.1337 | 0.3418 | 0.0394 |
| Shelter | *Usually* | 0.6910 | 0.3073 | 0.0867 |
| Shower or Bath | *Never* | 0.0357 | 0.1207 | 0.7800 |
| Shower or Bath | *Rarely* | 0.0088 | 0.1265 | 0.0501 |
| Shower or Bath | *Sometimes* | 0.0001 | 0.4082 | 0.0804 |
| Shower or Bath | *Usually* | 0.9553 | 0.3446 | 0.0896 |
| Average Classification Probabilities | | **1** | **2** | **3** |
| **Class 1** | | **0.8905** | 0.1093 | 0.0002 |
| **Class 2** | | 0.0244 | **0.9183** | 0.0573 |
| **Class 3** | | 0.0000 | 0.0893 | **0.9106** |
