# Supplementary material for: Material hardship, forced displacement, and negative health outcomes among unhoused people who use drugs in Los Angeles, California and Denver, Colorado: a latent class analysis
Source: BMC Public Health. 2025 Feb 13;25:591. doi: 10.1186/s12889-025-21626-6 (PMC11823192; doi:10.1186/s12889-025-21626-6)
Supplement: Supplementary file 2 — Supplementary Material 2. [file 12889_2025_21626_MOESM2_ESM.pdf]

Cannabis opioid qs -baseline for 3 and 6 (English)

Q1. **Id number** — — — — — —

Q2. **Site** (Choose one)

0	Denver
1	Los Angeles

Q3. **Interviewer initials (please record First, Middle, and Last Initial).** — — —

A. BACKGROUND

First, thank you for agreeing to be interviewed. I just want to emphasize that everything you tell me will be kept confidential. Your honesty is critical to the success of this project and will be important in understanding how to improve community health. We will start by asking a few questions about your background.

A1. **What is your date of birth?** __ __ / __ __ / __ __ __ __ mm / dd / yyyy

2097	Don't Know (Year)
2098	Refuse to Answer (Year)

A2. **How old are you?** — — — Age

997	Don't Know
998	Refuse to Answer
999	Not Applicable

SUBAGE = (TODAY - A1)/365

A3. **Are you male, female, transgender, or gender non-conforming?** (Choose one)

0	Male
1	Female
2	Transgender
3	Gender non-conforming
4	Other
7	Don't Know
8	Refuse to Answer

If A3 is less than 2, then skip to A5.

If A3 is greater than 2, then skip to A5.

A4. **Are you a male to female or female to male transgender person?** (Choose one)

0	Male to female
1	Female to male
7	Don't Know
8	Refuse to Answer
9	Not Applicable

A5. **Do you consider yourself to be Hispanic or Latino?** 1 Yes

0	No
7	Don't Know
8	Refuse to Answer
9	Not Applicable

A6. **What is your race? (check all that apply; do not read list)** (Check all that apply)

__	White
__	Black
__	Asian
__	Pacific Islander
__	Native American or Alaskan Native
__	Mixed race
__	Other
__	Don't Know
__	Refuse to Answer
__	Not Applicable

A7. **In the past month, how much money did you make from all sources, including legal and illegal sources? READ LIST** (Choose one)

0	Less than $1,000
1	$1,000 to $1,400
2	$1,401 to $2,100
3	$2,101 or more
7	Don't Know
8	Refuse to Answer

A8. **In the last <u>three months</u>, did you receive income from:**
(Read list aloud.) (Check all that apply)

Job you had	|__|
Unemployment	|__|
VA benefits	|__|
Welfare, food stamps, TANF, GA/GR	|__|
SSDI-Supplemental Security Disability Income or State disability	|__|
SSI or retirement benefits	|__|
Family and/or spouse	|__|
Recycling	|__|
Panhandling	|__|
Illegal or possibly illegal income	|__|
Friend	|__|
No income	|__|
Don't Know	|__|
Refuse to Answer	|__|

A9. **Did you graduate from high school or get a GED?** 1 Yes

0	No
7	Don't Know

8	Refuse to Answer
9	Not Applicable

A10. **Have you ever served in the US Armed Forces?** 1 Yes

0	No
7	Don't Know
8	Refuse to Answer
9	Not Applicable

A11. **What is your current relationship status? (Read list, choose one)** (Choose one)

0	Single, no main partner
1	In relationship but not living as married
2	Married or living as married
7	Don't Know
8	Refuse to Answer
9	Not Applicable

A12. **In the last 3 months, have you been homeless or unstably housed?**

Yes	1	
No	0	***Skip to A14***
Don't Know	7	
Refuse to Answer	8	

A13. **For how long have you been homeless or unstably housed? (Record as months - if less than a month enter 1) (That is, how long has it been since you were last stably housed.**)

__ __ __ __	Months
9997	Don't Know
9998	Refuse to Answer
9999	Not Applicable

A14. **What is the zip code of the area where you usually stay/sleep? (Refer to map if necessary) (If zipcode is unknown, please use the comment function (control-C) to record the street and nearest cross-street where the participant last slept.**)

__ __ __ __ __	Zip code
99997	Don't Know
99998	Refuse to Answer
99999	Not Applicable

A15. **In the last 3 months, let me know if you have stayed in any of the following types of places, even for one night?** (Check all that apply)

__	Tent
__	Outdoors (not in a tent)
__	Vehicle
__	Abandon building/garage/shed
__	Own house, apt, hotel room
__	Temporary hotel
__	Rented room in someone's place
__	With family, friend, girl/boyfriend
__	Shelter

__	Hospital
__	Jail or prison
__	Residential treatment
__	Group home
__	Don't Know
__	Refuse to Answer
__	Not Applicable

OUTDOOR = A15A + A15B + A15C + A15D

If OUTDOOR is equal to 0, then skip to instruction before A19.

A16. **In the last 3 months, how many nights did you stay in a tent, outdoors or in a car?**

__ __	Number
97	Don't Know
98	Refuse to Answer
99	Not Applicable

If A16 is less than 2, then skip to instruction before A19.

A17. **In the last 3 months, how many times did you move your tent, vehicle, or belongings to a different outdoor location (including across the street or down the block)?**

__ __ __	Moves
997	Don't Know
998	Refuse to Answer
999	Not Applicable

If A17 is equal to 0, then skip to instruction before A19.

A18. **In the last 3 months, how many of these moves were because of police, security guards, or other city/county officials?**

__ __	
97	Don't Know
98	Refuse to Answer
99	Not Applicable

If A17 is less than A18 then READ: "Your government induced moves were more than you total moves. Please go back 2 questions and clarify." and skip to instruction before A18.

If A12 is equal to 0, then skip to A22.

A19. **In the last 3 months, have you had any of your belongings taken or discarded by city, state, or county employees such as police or sanitation workers?**

1	Yes
0	No
7	Don't Know
8	Refuse to Answer
9	Not Applicable

If A19 is equal to 0, then skip to A21.

A20. **In the last 3 months, what kind of items were taken or discarded by governmental officials? (READ LIST)** (Check all that apply)

__	Identification

__ Other important paper work
__ Naloxone/Narcan
__ Suboxone/subutex (buprenorphine)
__ Chronic condition medication (e.g. diabetes)
__ Acute condition medication (e.g., bacterial infection)
__ Clothing
__ Food
__ Illicit drugs
__ Photos
__ Pet(s)
__ Syringes
__ Cotton or cookers (paraphernalia)
__ Biohazard containers
__ Tent or shelter
__ Don't Know
__ Refuse to Answer
__ Not Applicable

A21. **In the last 3 months, has any outreach worker or case manager offered you any kind of housing (shelter, transitional housing, permanent housing)? (We want to know if you were offered an actually housing slot that you could move into, rather than being put on a waiting list.)**

1 Yes
0 No
7 Don't Know
8 Refuse to Answer
9 Not Applicable

A22. **In the last 3 months, have you been evicted or asked to leave housing that you considered permanent or semi-permanent? An example of semi-permanent housing is a person who is in 6 month transitional housing and gets evicted at the end of the 6 months without obtaining new permanent housing.**

1 Yes
0 No
7 Don't Know
8 Refuse to Answer
9 Not Applicable

B. Substance use

The next questions are about your use of substances.

B1. **Have you ever injected drugs?** 1 Yes

0 No
7 Don't Know
8 Refuse to Answer
9 Not Applicable

If B1 is equal to 0, then skip to end of questionnaire.

B2. **The first time you injected drugs, how old were you?** __ __

Age

97	Don't Know
98	Refuse to Answer
99	Not Applicable

B3. **What was the first drug that you used by injection? (Do not read list)** (Choose one)

00	Crack
01	Powder Cocaine
02	Heroin
03	Fentanyl or other synthetic opioid
04	Prescription opiates like oxycontin
05	Methamphetamine or amphetamine
06	Speedball (heroin & cocaine combined)
07	Goofball (heroin & meth combined)
08	Prescription stimulant like ritalin
09	Prescription tranquilizer/benzodiazepines
10	Cannabis product
11	Methadone
12	Buprenorphine
13	Sedative/barbiturates
14	Ketamine/dissociative
15	Other
97	Don't Know
98	Refuse to Answer
99	Not Applicable

If B3 is less than 15, then skip to B5.

B4. **What was the other drug that you injected first?**

____

B5. **Had you used the first drug injected by some other route of use (for example, swallowed, snorted or smoked) before injecting it the first time?**

1	Yes
0	No
7	Don't Know
8	Refuse to Answer

If B5 is equal to 0, then skip to B8.

B6. **How had you taken this drug prior to injecting it? (Check all that apply)** (Check all that apply)

__	Smoked it
__	Sniffed or snorted it
__	Inhaled or "chased" it
__	Swallowed it
__	Inserted it rectally/anally
__	Other
__	Don't Know
__	Refuse to Answer

__	Not Applicable

If B6F is equal to 0, then skip to B8.

B7. **What other way did you use this drug?**

____

B8. **Have you used speedball in the last 3 months (a mixture of heroin and cocaine or crack)?**

1	Yes
0	No
7	Don't Know
8	Refuse to Answer
9	Not Applicable

If B8 is equal to 0, then skip to B13.

B9. **In the last 3 months, how many times have you used a speedball without injecting?**

__ __ __ __	Times
9997	Don't Know
9998	Refuse to Answer
9999	Not Applicable

If B9 is equal to 0, then skip to B12.

B10. **For your non-injection speedball use, how did you use or ingest this combination? Check all that apply** (Check all that apply)

__	Smoked (burned)
__	Sniffed or snorted by nose
__	Inhaled by mouth (heated)
__	Swallowed
__	Sublingual or dissolved under tongue
__	Rectal/Anal
__	Other
__	Don't Know
__	Refuse to Answer
__	Not Applicable

If B10G is equal to 0, then skip to B12.

B11. **What other method did you use for ingesting a speedball?**

____

B12. **In the last 3 months, how many times have you injected a speedball?**

__ __ __ __	Times
9997	Don't Know
9998	Refuse to Answer
9999	Not Applicable

B13. **Have you used goofball in the last 3 months (a mixture of heroin and methamphetamine)?**

1	Yes

0	No
7	Don't Know
8	Refuse to Answer
9	Not Applicable

If B13 is equal to 0, then skip to B18.

B14. **In the last 3 months, how many times have you used a goofball without injecting?**

__ __ __ __	Times
9997	Don't Know
9998	Refuse to Answer
9999	Not Applicable

If B14 is equal to 0, then skip to B17.

B15. **For your non-injection goofball use, how did you use or ingest this combination? Check all that apply** (Check all that apply)

__	Smoked (burned)
__	Sniffed or snorted by nose
__	Inhaled by mouth (heated)
__	Swallowed
__	Sublingual or dissolved under tongue
__	Rectal/Anal
__	Other
__	Don't Know
__	Refuse to Answer
__	Not Applicable

If B15G is equal to 0, then skip to B17.

B16. **What other method did you use for ingesting a goofball?**

__ __
__ __ __ __ __ __ __ __ __ __ __ __ __ __ __ __ __ __ __ __

B17. **In the last 3 months, how many times have you injected a goofball?**

__ __ __ __	Times
9997	Don't Know
9998	Refuse to Answer
9999	Not Applicable

B18. **Have you used crack or crack cocaine in the last 3 months?** 1 Yes

0	No
7	Don't Know
8	Refuse to Answer
9	Not Applicable

If B18 is equal to 0, then skip to B23.

B19. **In the last 3 months, how many times have you used crack without injecting?**

__ __ __ __	Times
9997	Don't Know

9998	Refuse to Answer
9999	Not Applicable

If B19 is equal to 0, then skip to B22.

B20. **For your non-injection crack use, how did you use or ingest this drug? Check all that apply** (Check all that apply)

- __ Smoked (burned)
- __ Sniffed or snorted by nose
- __ Inhaled by mouth (heated)
- __ Swallowed
- __ Sublingual or dissolved under tongue
- __ Rectal/Anal
- __ Other
- __ Don't Know
- __ Refuse to Answer
- __ Not Applicable

If B20G is equal to 0, then skip to B22.

B21. **What other method did you use for ingesting a crack?**

———
————————————————————

B22. **In the last 3 months, how many times have you injected crack?** __ __ __ __ Times

9997	Don't Know
9998	Refuse to Answer
9999	Not Applicable

B23. **Have you used powder cocaine in the last 3 months?** 1 Yes

0	No
7	Don't Know
8	Refuse to Answer
9	Not Applicable

If B23 is equal to 0, then skip to B28.

B24. **In the last 3 months, how many times have you used powder cocaine without injecting?**

__ __ __ __	Times
9997	Don't Know
9998	Refuse to Answer
9999	Not Applicable

If B24 is equal to 0, then skip to B27.

B25. **For your non-injection powder cocaine use, how did you use or ingest this drug?** (Check all that apply)

- __ Smoked (burned)
- __ Sniffed or snorted by nose
- __ Inhaled by mouth (heated)
- __ Swallowed

__	Sublingual or dissolved under tongue
__	Rectal/Anal
__	Other
__	Don't Know
__	Refuse to Answer
__	Not Applicable

If B25G is equal to 0, then skip to B27.

B26. **What other method did you use for ingesting powder cocaine?**

— —
— — — — — — — — — — — — — — — — — — — —

B27. **In the last 3 months, how many times have you injected powder cocaine?**

— — — —	Times
9997	Don't Know
9998	Refuse to Answer
9999	Not Applicable

B28. **Have you used methamphetamine by itself in the last 3 months?**

1	Yes
0	No
7	Don't Know
8	Refuse to Answer
9	Not Applicable

If B28 is equal to 0, then skip to B33.

B29. **In the last 3 months, how many times have you used methamphetamine by itself without injecting?**

— — — —	Times
9997	Don't Know
9998	Refuse to Answer
9999	Not Applicable

If B29 is equal to 0, then skip to B32.

B30. **For your non-injection methamphetamine use, how did you use or ingest this drug?** (Check all that apply)

__	Smoked (burned)
__	Sniffed or snorted by nose
__	Inhaled by mouth (heated)
__	Swallowed
__	Sublingual or dissolved under tongue
__	Rectal/Anal
__	Other
__	Don't Know
__	Refuse to Answer
__	Not Applicable

If B30G is equal to 0, then skip to B32.

B31. **What other method did you use for ingesting methamphetamine?**

— —

— —

B32. **In the last 3 months, how many times have you injected methamphetamine by itself?**

__ __ __ __	Times
9997	Don't Know
9998	Refuse to Answer
9999	Not Applicable

B33. **Have you used heroin by itself in the last 3 months?**

1	Yes
0	No
7	Don't Know
8	Refuse to Answer
9	Not Applicable

If B33 is equal to 0, then skip to B38.

B34. **In the last 3 months, how many times have you used heroin without injecting?**

__ __ __ __	Times
9997	Don't Know
9998	Refuse to Answer
9999	Not Applicable

If B34 is equal to 0, then skip to B37.

B35. **For your non-injection heroin use, how did you use or ingest this drug?** (Check all that apply)

__ Smoked (burned)
__ Sniffed or snorted by nose
__ Inhaled by mouth (heated)
__ Swallowed
__ Sublingual or dissolved under tongue
__ Rectal/Anal
__ Other
__ Don't Know
__ Refuse to Answer
__ Not Applicable

If B35G is equal to 0, then skip to B37.

B36. **What other method did you use for ingesting heroin?**

— —
— —

B37. **In the last 3 months, how many times have you injected heroin by itself?**

__ __ __ __	Times
9997	Don't Know
9998	Refuse to Answer
9999	Not Applicable

B38. **Have you used fentanyl or another synthetic opioid (such as carfentanil) without a prescription in the last 3 months by itself? (Include synthetic opioids that were licitly (obtained from hospital or pharmacy) and illicitly manufactured drug.)**

1	Yes
0	No
7	Don't Know
8	Refuse to Answer
9	Not Applicable

If B38 is equal to 0, then skip to instruction before B43.

B39. **In the last 3 months, how many times have you used fentanyl without injecting by itself?**

__ __ __ __	Times
9997	Don't Know
9998	Refuse to Answer
9999	Not Applicable

If B39 is equal to 0, then skip to B42.

B40. **For your non-injection fentanyl use, how did you use or ingest it? Check all that apply** (Check all that apply)

- __ Smoked it
- __ Sniffed or snorted it
- __ Inhaled or "chased" it
- __ Swallowed it
- __ Inserted it rectally
- __ Used as patch
- __ Other
- __ Don't Know
- __ Refuse to Answer
- __ Not Applicable

If B40G is equal to 0, then skip to B42.

B41. **What other method did you use for ingesting fentanyl?**

__ __
__ __ __ __ __ __ __ __ __ __ __ __ __ __ __ __ __ __ __ __

B42. **In the last 3 months, how many times have you injected fentanyl by itself?**

__ __ __ __	Times
9997	Don't Know
9998	Refuse to Answer
9999	Not Applicable

The next set of questions is about drugs that are typically prescribed by physicians. We are interested in your use of these medications without a doctor's prescription or your use of these medications not as directed by your physician.

B43. **Have you used an opioid (such as tramadol, vicodin, oxycontin, opana, dilaudid, percocet, oxycodone, or morphine) without a prescription in the last 3 months? (Do not include synthetic opioids like fentanyl or carfentanil**)

1	Yes
0	No
7	Don't Know
8	Refuse to Answer

9	Not Applicable

If B43 is equal to 0, then skip to B48.

B44. **In the last 3 months, how many times have you used an opioid without injecting?**

— — — —	Times
9997	Don't Know
9998	Refuse to Answer
9999	Not Applicable

If B44 is equal to 0, then skip to B47.

B45. **For your non-injection prescription opioid use, how did you use or ingest this drug?** (Check all that apply)

__	Smoked it
__	Sniffed or snorted it
__	Inhaled or "chased" it
__	Swallowed it
__	Inserted it rectally
__	Other
__	Don't Know
__	Refuse to Answer
__	Not Applicable

If B45F is equal to 0, then skip to B47.

B46. **What other method did you use for ingesting opioid prescription medications?**

— —
— — — — — — — — — — — — — — — — — — — —

B47. **In the last 3 months, how many times have you injected an opioid?**

— — — —	Times
9997	Don't Know
9998	Refuse to Answer
9999	Not Applicable

B48. **Have you used a tranquilizer/benzodiazepine (such as klonopin, Xanax, valium, or ativan) without a prescription in the last 3 months?** (Or research chemicals such as clonzolam, bromazolam, and flualprazolam)

1	Yes
0	No
7	Don't Know
8	Refuse to Answer
9	Not Applicable

If B48 is equal to 0, then skip to B53.

B49. **In the last 3 months, how many times have you used a tranquilizer/benzodiazepine without injecting?**

— — — —	Times
9997	Don't Know
9998	Refuse to Answer

9999	Not Applicable

If B49 is equal to 0, then skip to B52.

B50. **For your non-injection tranquilizer/benzodiazepine use, how did you use or ingest it? Check all that apply** (Check all that apply)

__	Smoked (burned)
__	Sniffed or snorted by nose
__	Inhaled by mouth (heated)
__	Swallowed
__	Sublingual or dissolved under tongue
__	Rectal/Anal
__	Other
__	Don't Know
__	Refuse to Answer
__	Not Applicable

If B50G is equal to 0, then skip to B52.

B51. **What other method did you use for ingesting a TRANQUILIZER/benzodiazepine?**

____
____

B52. **In the last 3 months, how many times have you injected tranquilizers/benzodiazepines?**

____	Times
9997	Don't Know
9998	Refuse to Answer
9999	Not Applicable

B53. **Have you used a sedative (such as restoril, phenobarbital, placidyl, ambien, Lunesta, gabapentin) without a prescription in the last 3 months?**

1	Yes
0	No
7	Don't Know
8	Refuse to Answer
9	Not Applicable

If B53 is equal to 0, then skip to B58.

B54. **In the last 3 months, how many times have you used a sedative without injecting?**

____	Times
9997	Don't Know
9998	Refuse to Answer
9999	Not Applicable

If B54 is equal to 0, then skip to B57.

B55. **For your non-injection sedative use, how did you use or ingest it? Check all that apply** (Check all that apply)

__	Smoked (burned)
__	Sniffed or snorted by nose
__	Inhaled by mouth (heated)

__	Swallowed
__	Sublingual or dissolved under tongue
__	Rectal/Anal
__	Other
__	Don't Know
__	Refuse to Answer
__	Not Applicable

If B55G is equal to 0, then skip to B57.

B56. **What other method did you use for ingesting a sedative?**

____

B57. **In the last 3 months, how many times have you injected a sedative?**

__ __ __ __	Times
9997	Don't Know
9998	Refuse to Answer
9999	Not Applicable

B58. **Have you used a stimulant (such as ritalin, aderall, methedrine, benzedrine) without a prescription in the last 3 months?**

1	Yes
0	No
7	Don't Know
8	Refuse to Answer
9	Not Applicable

If B58 is equal to 0, then skip to B63.

B59. **In the last 3 months, how many times have you used a stimulant without injecting?**

__ __ __ __	Times
9997	Don't Know
9998	Refuse to Answer
9999	Not Applicable

If B59 is equal to 0, then skip to B62.

B60. **For your non-injection stimulant use, how did you use or ingest it? Check all that apply** (Check all that apply)

__	Smoked (burned)
__	Sniffed or snorted by nose
__	Inhaled by mouth (heated)
__	Swallowed
__	Sublingual or dissolved under tongue
__	Rectal/Anal
__	Other
__	Don't Know
__	Refuse to Answer
__	Not Applicable

If B60G is equal to 0, then skip to B62.

B61. **What other method did you use for ingesting a stimulant?**

__ __
__ __ __ __ __ __ __ __ __ __ __ __ __ __ __ __ __ __ __ __

B62. **In the last 3 months, how many times have you injected a stimulant?**

__ __ __ __	Times
9997	Don't Know
9998	Refuse to Answer
9999	Not Applicable

B63. **Have you used a methadone without a prescription in the last 3 months?** 1 Yes

0	No
7	Don't Know
8	Refuse to Answer
9	Not Applicable

If B63 is equal to 0, then skip to B68.

B64. **In the last 3 months, how many times have you used methadone without injecting?**

__ __ __ __	Times
9997	Don't Know
9998	Refuse to Answer
9999	Not Applicable

If B64 is equal to 0, then skip to B67.

B65. **For your non-injection methadone use, how did you use or ingest it? Check all that apply** (Check all that apply)

__ Smoked (burned)
__ Sniffed or snorted by nose
__ Inhaled by mouth (heated)
__ Swallowed
__ Sublingual or dissolved under tongue
__ Rectal/Anal
__ Other
__ Don't Know
__ Refuse to Answer
__ Not Applicable

If B65G is equal to 0, then skip to B67.

B66. **What other method did you use for ingesting methadone?**

__ __
__ __ __ __ __ __ __ __ __ __ __ __ __ __ __ __ __ __ __ __

B67. **In the last 3 months, how many times have you injected methadone?**

__ __ __ __	Times
9997	Don't Know
9998	Refuse to Answer
9999	Not Applicable

B68. **Have you used buprenorphine, suboxone, or subutex without a prescription in the last 3 months?**

1	Yes
0	No
7	Don't Know
8	Refuse to Answer
9	Not Applicable

If B68 is equal to 0, then skip to instruction before B73.

B69. **In the last 3 months, how many times have you used buprenorphine without injecting?**

__ __ __ __	Times
9997	Don't Know
9998	Refuse to Answer
9999	Not Applicable

If B69 is equal to 0, then skip to B72.

B70. **For your non-injection buprenorphine use, how did you use or ingest it? Check all that apply** (Check all that apply)

__	Smoked (burned)
__	Sniffed or snorted by nose
__	Inhaled by mouth (heated)
__	Swallowed
__	Sublingual or dissolved under tongue
__	Rectal/Anal
__	Other
__	Don't Know
__	Refuse to Answer
__	Not Applicable

If B70G is equal to 0, then skip to B72.

B71. **What other method did you use for ingesting buprenorphine?**

__ __

B72. **In the last 3 months, how many times have you injected buprenorphine?**

__ __ __ __	Times
9997	Don't Know
9998	Refuse to Answer
9999	Not Applicable

SUMINJ = B12 + B17 + B22 + B27 + B32 + B37 + B47 + B42 + B52 + B57 + B62 + B67 + B72

HERSUM = B9 + B12 + B14 + B17 + B34 + B37 + B44 + B47 + B39 + B42

COKSUM = B9 + B12 + B19 + B22 + B24 + B27

METSUM = B14 + B17 + B29 + B32

OSTSUM = B64 + B67 + B69 + B72

NUMOPIOID = B8 + B13 + B33 + B43 + B38

If NUMOPIOID is equal to 0, then skip to end of questionnaire.

The next questions are about your use of marijuana and/or cannabis and cannabis products. We are interested in your use of marijuana/cannabis regardless of whether your use is for medical or non-medical purposes.

B73. **Have you ever used marijuana or cannabis?**

1	Yes
0	No
7	Don't Know
8	Refuse to Answer
9	Not Applicable

If B73 is equal to 0, then skip to instruction before B116.

B74. **How old were you when you first used marijuana or cannabis?**

__ __	Age
97	Don't Know
98	Refuse to Answer
99	Not Applicable

B75. **Have you used marijuana or cannabis in the last 3 months?**

1	Yes
0	No
7	Don't Know
8	Refuse to Answer
9	Not Applicable

If B75 is equal to 0, then skip to instruction before B116.

B76. **In the last 3 months, how many times have you used marijuana or cannabis?**

__ __ __ __	Times
9997	Don't Know
9998	Refuse to Answer
9999	Not Applicable

B77. **For your marijuana and cannabis use in the past 3 months, how did you use or ingest it? (DO NOT READ LIST)** (Check all that apply)

__	Smoked (burned/lit)
__	Vaped (heated)
__	Ate (Edibles)
__	Dab rig for concentrates/budder/badder
__	Topicals (cream, salve, oils)
__	Other oral use (tinctures, oils, capsules)
__	Injection
__	Other
__	Don't Know
__	Refuse to Answer
__	Not Applicable

If B77H is equal to 0, then skip to B79.

B78. **What other method did you use for ingesting marijuana or cannabis?**

———

B79. **How have you mostly used cannabis?** (Choose one)

00	Smoked
01	Vaped
02	Ate
03	Dab rig (concentrate)
04	Topical cream/salve
05	Other oral
06	Injection
07	Other
97	Don't Know
98	Refuse to Answer
99	Not Applicable

SUMNON = B9 + B14 + B19 + B24 + B29 + B34 + B44 + B39 + B49 + B54 + B59 + B64 + B69 + B76

B80. **Do you have a medical marijuana/cannabis card or a referral letter for medical use of marijuana or cannabis?**

1	Yes
0	No
7	Don't Know
8	Refuse to Answer
9	Not Applicable

B81. **From which of the following sources have you obtained cannabis or marijuana from in the last 3 months? (Check all that apply)** (Check all that apply)

__ Friend or acquaintance
__ Family
__ Dealer (not dispensary)
__ Medical dispensary
__ Recreational dispensary
__ Substance use treatment program
__ Grew my own
__ Received from cannabis distribution program
__ Other
__ Don't Know
__ Refuse to Answer
__ Not Applicable

If B81I is equal to 0, then skip to B83.

B82. **What was this other source?**

____

B83. **From which source did you obtain most of the cannabis you used in the last 3 months?** (Choose one)

00	Friend or acquaintance
01	Family
02	Street dealer

03	Medical dispensary
04	Recreational dispensary
05	Substance use treatment clinic
06	Grew my own
07	Free cannabis distribution
08	Other
97	Don't Know
98	Refuse to Answer
99	Not Applicable

B84. **Which types of cannabis have you used in the last 3 months? (Read list)** (Check all that apply)

__	Indica flower
__	Sativa flower
__	Hybrid
__	High CBD
__	High THC
__	Concentrate
__	Other
__	Don't Know
__	Refuse to Answer
__	Not Applicable

If B84G is equal to 0, then skip to B86.

If B84G is equal to 97, then skip to B87.

B85. **What is this other type of cannabis that you used?**

— —

B86. **Which type of cannabis did you use the most in the last 3 months** (Choose one)

0	Indica
1	Sativa
2	Hybrid
3	High CBD
4	High THC
5	Concentrate
6	Other
7	Don't Know
8	Refuse to Answer
9	Not Applicable

B87. **Have you purchased cannabis/marijuana products in the last 3 months?**

1	Yes
0	No
7	Don't Know
8	Refuse to Answer
9	Not Applicable

If B87 is equal to 0, then skip to instruction before B97.

If B81C is equal to 0, then skip to instruction before B91.

B88. **The last time you purchased cannabis from a source other than a dispensary, how much did the cannabis cost per gram? (dollars per gram) (For interviewer only: A dime bag of cannabis is usually a half gram and cost $10. So the gram cost of a dime bag in this example is $20. There are 28 grams in an ounce. Therefore, a 1/4 ounce is 7 grams. A typical joint is a third of a gram. So if a joint cost a dollar than the price per gram was $3.**)

$__ __ __ __	Dollars
9997	Don't Know
9998	Refuse to Answer
9999	Not Applicable

B89. **Was the amount you paid to this non-dispensary source more, less or about the same as you would usually spend for the same amount of cannabis/marijuana from this source?** (Choose one)

0	Less
1	About the same
2	More
3	Don't purchase MJ from this source
7	Don't Know
8	Refuse to Answer
9	Not Applicable

B90. **How would you rate the quality of the cannabis/marijuana you last purchased from the dealer?** (Choose one)

0	Low grade
1	Medium grade
2	High grade
7	Don't Know
8	Refuse to Answer
9	Not Applicable

If B81D is equal to 0, then skip to instruction before B94.

B91. **The last time you purchased cannabis from a medical dispensary, how much did the cannabis cost per gram? (Dollars per gram) (For interviewer only: A dime bag of cannabis is usually a half gram and cost $10. So the gram cost of a dime bag in this example is $20. There are 28 grams in an ounce. Therefore, a 1/4 ounce is 7 grams. A typical joint is a third of a gram. So if a joint cost a dollar than the price per gram was $3.**
)

$__ __ __ __	Dollars
9997	Don't Know
9998	Refuse to Answer
9999	Not Applicable

B92. **Was the amount you paid to the medical dispensary more, less or about the same as you would usually spend for the same amount of cannabis/marijuana from this source?** (Choose one)

0	Less
1	About the same
2	More
3	Don't purchase MJ from this source
7	Don't Know
8	Refuse to Answer
9	Not Applicable

B93. **How would you rate the quality of the cannabis/marijuana you last purchased from the medical dispensary?** (Choose one)

0	Low grade
1	Medium grade
2	High grade
7	Don't Know
8	Refuse to Answer
9	Not Applicable

If B81E is equal to 0, then skip to instruction before B97.

B94. **The last time you purchased cannabis from a recreational dispensary, how much did the cannabis cost per gram? (Dollars per gram) (For interviewer only: A dime bag of cannabis is usually a half gram and cost $10. So the gram cost of a dime bag in this example is $20. There are 28 grams in an ounce. Therefore, a 1/4 ounce is 7 grams. A typical joint is a third of a gram. So if a joint cost a dollar than the price per gram was $3.**
)

$__ __ __ __	Dollars
9997	Don't Know
9998	Refuse to Answer
9999	Not Applicable

B95. **Was the amount you paid to the recreational dispensary more, less or about the same as you would usually spend for the same amount of cannabis/marijuana from this source?** (Choose one)

0	Less
1	About the same
2	More
3	Don't purchase MJ from this source
7	Don't Know
8	Refuse to Answer
9	Not Applicable

B96. **How would you rate the quality of the cannabis/marijuana you last purchased from the recreational dispensary?** (Choose one)

0	Low grade
1	Medium grade
2	High grade
7	Don't Know
8	Refuse to Answer
9	Not Applicable

Many people drink alcohol when using cannabis. For the next 4 questions, we want to know how often you combine cannabis use with use of other substances.

B97. **When you used marijuana or cannabis in the last 3 months, how often did you use it with alcohol?** (Choose one)

0	None
1	Some
2	About half
3	Most
4	All

7	Don't Know
8	Refuse to Answer
9	Not Applicable

B98. **When you used marijuana or cannabis in the last 3 months, how often did you use it with tobacco or nicotine?** (Choose one)

0	None
1	Some
2	About half
3	Most
4	All
7	Don't Know
8	Refuse to Answer
9	Not Applicable

B99. **When you used marijuana or cannabis in the last 3 months, how often did you use it with substances like heroin, methamphetamine, crack cocaine, cocaine?** (Choose one)

0	None
1	Some
2	About half
3	Most
4	All
7	Don't Know
8	Refuse to Answer
9	Not Applicable

If B99 is equal to 0, then skip to instruction before B103.

B100. **What other drug have you used the most with cannabis in the last 3 months? (Do not read list)** (Choose one)

00	Crack
01	Powder Cocaine
02	Heroin
03	Fentanyl or other synthetic opioid
04	Prescription opiates like oxycontin
05	Methamphetamine or amphetamine
06	Speedball (heroin & cocaine combined)
07	Goofball (heroin & meth combined)
08	Prescription stimulant like ritalin
09	Prescription tranquilizer/benzodiazepines
10	Methadone
11	Buprenorphine
12	Sedative/barbiturates
13	Other
97	Don't Know
98	Refuse to Answer
99	Not Applicable

B101. **In the last 3 months, when you have mixed cannabis with drugs like heroin, prescription opiates, methamphetamine, and cocaine, did you do this for any of the following reasons?** (Check all that

apply)

__	To get a better high
__	To reduce the use of either or both substances
__	Because you had both
__	To modify or manage the high
__	To manage the come down
__	Other
__	Don't Know
__	Refuse to Answer
__	Not Applicable

If B101F is equal to 0, then skip to instruction before B103.

B102. **What were your other reasons for using cannabis with other substances?**

———————————————————————————————————————

The next set of questions are about your reasons for using cannabis in general.

B103. **When you used marijuana or cannabis in the last 3 months, how often did you use it to relieve physical pain?** (Choose one)

0	None
1	Some
2	About half
3	Most
4	All
7	Don't Know
8	Refuse to Answer
9	Not Applicable

B104. **When you used marijuana or cannabis in the last 3 months, how often did you use it to relieve opioid or heroin withdrawal symptoms and/or pain?** (Choose one)

0	None
1	Some
2	About half
3	Most
4	All
7	Don't Know
8	Refuse to Answer
9	Not Applicable

B105. **When you used marijuana or cannabis in the last 3 months, how often did you use it to relieve emotional problems such as depression, anxiety, or feelings of sadness?** (Choose one)

0	None
1	Some
2	About half
3	Most
4	All
7	Don't Know
8	Refuse to Answer
9	Not Applicable

B106. **When you used marijuana or cannabis in the last 3 months, how often did you use it to reduce your heroin, fentanyl, or prescription opiate use?** (Choose one)

0	None
1	Some
2	About half
3	Most
4	All
7	Don't Know
8	Refuse to Answer
9	Not Applicable

B107. **When you used marijuana or cannabis in the last 3 months, how often did you use it to reduce your alcohol use?** (Choose one)

0	None
1	Some
2	About half
3	Most
4	All
7	Don't Know
8	Refuse to Answer
9	Not Applicable

B108. **When you used marijuana or cannabis in the last 3 months, how often did you use it to reduce your stimulant or methamphetamine use?** (Choose one)

0	None
1	Some
2	About half
3	Most
4	All
7	Don't Know
8	Refuse to Answer
9	Not Applicable

B109. **When you used marijuana or cannabis in the last 3 months, how often did you use it to help with sleep?** (Choose one)

0	None
1	Some
2	About half
3	Most
4	All
7	Don't Know
8	Refuse to Answer
9	Not Applicable

B110. **When you used marijuana or cannabis in the last 3 months, how often did you use it to treat nausea or loss of appetite?** (Choose one)

0	None
1	Some
2	About half

3 Most
4 All
7 Don't Know
8 Refuse to Answer
9 Not Applicable

B111. **When you used marijuana or cannabis in the last 3 months, how often did you use it to get high?** (Choose one)

0 None
1 Some
2 About half
3 Most
4 All
7 Don't Know
8 Refuse to Answer
9 Not Applicable

B112. **Have you <u>EVER</u> used cannabis/marijuana to reduce your use of other substances such as alcohol, tobacco, heroin, prescription opiates, etc?**

1 Yes
0 No
7 Don't Know
8 Refuse to Answer
9 Not Applicable

If B112 is equal to 0, then skip to instruction before B116.

B113. **Which drugs were you trying to reduce when you used cannabis?** (Check all that apply)

__ Alcohol
__ Tobacco or nicotine
__ Crack cocaine
__ Powder cocaine
__ Heroin
__ Fentanyl
__ Prescription opiates
__ Methamphetamine
__ Speedball
__ Goofball
__ Prescription stimulants
__ Prescription tranquilizers
__ Prescription sedatives
__ Methadone
__ Buprenorphine
__ Don't Know
__ Refuse to Answer
__ Not Applicable

B114. **Did it work for any drug?** 1 Yes

0 No

7	Don't Know
8	Refuse to Answer
9	Not Applicable

B115. **For which drug did using cannabis/marijuana result in a reduction in use?** (Check all that apply)

__	Alcohol
__	Tobacco or nicotine
__	Crack cocaine
__	Powder cocaine
__	Heroin
__	Fentanyl
__	Prescription opiates
__	Methamphetamine
__	Speedball
__	Goofball
__	Prescription stimulants
__	Prescription tranquilizers
__	Prescription sedatives
__	Methadone
__	Buprenorphine
__	Don't Know
__	Refuse to Answer
__	Not Applicable

The next set of questions are about your use of alcohol and tobacco.

B116. **In the last 3 months, on how many days did you drink a beverage containing alcohol?**

__ __	Days
97	Don't Know
98	Refuse to Answer
99	Not Applicable

If B116 is equal to 0, then skip to B119.

For the next two items, please note that a standard alcohol drink is equal to 12 ounces of regular beer, 8 to 9 ounces of malt liquor beer, 5 ounces of wine, 3 to 4 ounces of fortified wine, brandy, or port, and 1.5 ounces of hard liquor (or a shot).

B117. **During the last 3 months, on a typical day when you drank alcohol, how many standard drinks did you have?**

__ __ __	Drinks
997	Don't Know
998	Refuse to Answer
999	Not Applicable

B118. **In the last 3 months, what is the largest number of standard drinks you had on any single day?**

__ __	drinks
97	Don't Know
98	Refuse to Answer
99	Not Applicable

B119. **Do you currently smoke cigarettes? (Limit to combustible tobacco including cigars and pipes, but not e-cigarettes).**

1	Yes
0	No
7	Don't Know
8	Refuse to Answer
9	Not Applicable

If B119 is equal to 0, then skip to instruction before B122.

B120. **How many cigarettes do you smoke a day on average? (FYI, a pack has 20 cigarettes).**

— — —	# of cigarettes
997	Don't Know
998	Refuse to Answer
999	Not Applicable

B121. **How many years have you smoked?** — — Years

97	Don't Know
98	Refuse to Answer
99	Not Applicable

If B119 is equal to 1, then skip to instruction before B125.

B122. **Have you ever smoked cigarettes?** 1 Yes

0	No
7	Don't Know
8	Refuse to Answer
9	Not Applicable

If B122 is equal to 0, then skip to instruction before B125.

B123. **How many years did you smoke? (If less than a year, enter 1).** — — Years

97	Don't Know
98	Refuse to Answer
99	Not Applicable

B124. **How many packs a day did you smoke? (If less than 1 pack, enter 1) (a pack has 20 cigarettes).**

— — —	
997	Don't Know
998	Refuse to Answer
999	Not Applicable

The next questions are about whether fentanyl is available in illicit drug markets where you live and/or hang out. I am interested in fentanyl that you have obtained on the streets or illicitly and NOT that was prescribed to you.

B125. **Is fentanyl (in any form) available in the area where you live and/or spend most of your time? (Any form includes only or mixed intentionally or unintentionally in other substances.)**

1	Yes

0	No
7	Don't Know
8	Refuse to Answer
9	Not Applicable

If B125 is equal to 0, then skip to B127.

B126. **In what form is fentanyl available? (READ LIST)** (Check all that apply)

__ Mixed with heroin/sold as mixed
__ Mixed with heroin/sold as heroin
__ Sold as fentanyl
__ Sold as heroin, but is only fentanyl
__ Sold as counterfeit pills w/out fentanyl
__ Sold as counterfeit pills with fentanyl
__ Don't Know
__ Refuse to Answer
__ Not Applicable

B127. **In the last 3 month, have you used any drug that you suspected or thought was mixed with fentanyl or some other synthetic opioid?**

1	Yes
0	No
7	Don't Know
8	Refuse to Answer
9	Not Applicable

If B127 is equal to 0, then skip to B143.

B128. **Please select the drugs that you used in the last 3 months that were mixed with fentanyl or other synthetic opioids?** (Check all that apply)

__ Crack
__ Powder cocaine
__ Heroin
__ Prescription Opiates
__ Methamphetamine
__ Speedball
__ Goofball
__ Stimulants
__ Tranquilizers
__ Cannabis
__ Methadone
__ Buprenorphine
__ Sedative
__ Ketamine/dissociative
__ Don't Know
__ Refuse to Answer
__ Not Applicable ***Skip to B143***

If B128A is equal to 0, then skip to instruction before B130.

B129. **In the last 3 months, how frequently was the crack that you used mixed with fentanyl?** (Choose

one)

0	Never - 0%
1	Occasionally - 1% to 25%
2	Sometimes - 26% to 74%
3	Usually - 75% to 99%
4	Always - 100%
7	Don't Know
8	Refuse to Answer
9	Not Applicable

If B128B is equal to 0, then skip to instruction before B131.

B130. **In the last 3 months, how frequently was the powder cocaine that you used mixed with fentanyl?** (Choose one)

0	Never - 0%
1	Occasionally - 1% to 25%
2	Sometimes - 26% to 74%
3	Usually - 75% to 99%
4	Always - 100%
7	Don't Know
8	Refuse to Answer
9	Not Applicable

If B128C is equal to 0, then skip to instruction before B132.

B131. **In the last 3 months, how frequently was the heroin you used mixed with fentanyl?** (Choose one)

0	Never - 0%
1	Occasionally - 1% to 25%
2	Sometimes - 26% to 74%
3	Usually - 75% to 99%
4	Always - 100%
7	Don't Know
8	Refuse to Answer
9	Not Applicable

If B128D is equal to 0, then skip to instruction before B133.

B132. **In the last 3 months, how frequently was the prescription opioids that you used mixed with fentanyl?** (Choose one)

0	Never - 0%
1	Occasionally - 1% to 25%
2	Sometimes - 26% to 74%
3	Usually - 75% to 99%
4	Always - 100%
7	Don't Know
8	Refuse to Answer
9	Not Applicable

If B128E is equal to 0, then skip to instruction before B134.

B133. **In the last 3 months, how frequently was the methamphetamine that you used mixed with fentanyl?**

(Choose one)

0	Never - 0%
1	Occasionally - 1% to 25%
2	Sometimes - 26% to 74%
3	Usually - 75% to 99%
4	Always - 100%
7	Don't Know
8	Refuse to Answer
9	Not Applicable

If B128F is equal to 0, then skip to instruction before B135.

B134. **In the last 3 months, how frequently were the speedballs that you used mixed with fentanyl?** (Choose one)

0	Never - 0%
1	Occasionally - 1% to 25%
2	Sometimes - 26% to 74%
3	Usually - 75% to 99%
4	Always - 100%
7	Don't Know
8	Refuse to Answer
9	Not Applicable

If B128G is equal to 0, then skip to instruction before B136.

B135. **In the last 3 months, how frequently were the goofballs that you used mixed with fentanyl?** (Choose one)

0	Never - 0%
1	Occasionally - 1% to 25%
2	Sometimes - 26% to 74%
3	Usually - 75% to 99%
4	Always - 100%
7	Don't Know
8	Refuse to Answer
9	Not Applicable

If B128H is equal to 0, then skip to instruction before B137.

B136. **In the last 3 months, how frequently were the prescription stimulants that you used mixed with fentanyl?** (Choose one)

0	Never - 0%
1	Occasionally - 1% to 25%
2	Sometimes - 26% to 74%
3	Usually - 75% to 99%
4	Always - 100%
7	Don't Know
8	Refuse to Answer
9	Not Applicable

If B128I is equal to 0, then skip to instruction before B138.

B137. **In the last 3 months, how frequently were the prescription tranquilizers that you used mixed with fentanyl?** (Choose one)

0	Never - 0%
1	Occasionally - 1% to 25%
2	Sometimes - 26% to 74%
3	Usually - 75% to 99%
4	Always - 100%
7	Don't Know
8	Refuse to Answer
9	Not Applicable

If B128J is equal to 0, then skip to instruction before B139.

B138. **In the last 3 months, how frequently was the cannabis that you used mixed with fentanyl?** (Choose one)

0	Never - 0%
1	Occasionally - 1% to 25%
2	Sometimes - 26% to 74%
3	Usually - 75% to 99%
4	Always - 100%
7	Don't Know
8	Refuse to Answer
9	Not Applicable

If B128K is equal to 0, then skip to instruction before B140.

B139. **In the last 3 months, how frequently was the methadone that you used mixed with fentanyl?** (Choose one)

0	Never - 0%
1	Occasionally - 1% to 25%
2	Sometimes - 26% to 74%
3	Usually - 75% to 99%
4	Always - 100%
7	Don't Know
8	Refuse to Answer
9	Not Applicable

If B128L is equal to 0, then skip to instruction before B141.

B140. **In the last 3 months, how frequently was the buprenorphine/suboxone that you used mixed with fentanyl?** (Choose one)

0	Never - 0%
1	Occasionally - 1% to 25%
2	Sometimes - 26% to 74%
3	Usually - 75% to 99%
4	Always - 100%
7	Don't Know
8	Refuse to Answer
9	Not Applicable

If B128M is equal to 0, then skip to instruction before B142.

B141. **In the last 3 months, how frequently were the prescription sedatives that you used mixed with fentanyl?** (Choose one)

0	Never - 0%
1	Occasionally - 1% to 25%
2	Sometimes - 26% to 74%
3	Usually - 75% to 99%
4	Always - 100%
7	Don't Know
8	Refuse to Answer
9	Not Applicable

If B128N is equal to 0, then skip to B143.

B142. **In the last 3 months, how frequently was the ketamine/dissociative that you used mixed with fentanyl?** (Choose one)

0	Never - 0%
1	Occasionally - 1% to 25%
2	Sometimes - 26% to 74%
3	Usually - 75% to 99%
4	Always - 100%
7	Don't Know
8	Refuse to Answer
9	Not Applicable

B143. **Have you ever used a fentanyl test strip on drugs you were about to use? (either injected or non-injected).**

1	Yes
0	No
7	Don't Know
8	Refuse to Answer
9	Not Applicable

If B143 is equal to 0, then skip to instruction before C1.

B144. **How many times have use ever used a fentanyl test strip to check drugs you were about to use?**

— — — — —	Times
99997	Don't Know
99998	Refuse to Answer
99999	Not Applicable

B145. **Have you used a fentanyl test strip in the last 3 months?**

1	Yes
0	No
7	Don't Know
8	Refuse to Answer
9	Not Applicable

B146. **The last time you used a fentanyl test strip, was there fentanyl in the sample?**

1	Yes

0	No
7	Don't Know
8	Refuse to Answer
9	Not Applicable

If B146 is equal to 0, then skip to B148.

B147. **What did you do following the positive fentanyl test result?** (Check all that apply)

__ Used as intended
__ Used less than intended
__ Pushed plunger more slowly than usual
__ Pushed plunger partway and waited
__ Sniffed/tooted instead of injecting
__ Threw away drug
__ Sold the drug
__ Shared test results with other people
__ Don't Know
__ Refuse to Answer
__ Not Applicable

B148. **Does using a fentanyl test strip make you feel better able to protect yourself from overdose?**

1	Yes
0	No
7	Don't Know
8	Refuse to Answer
9	Not Applicable

C. Syringe Access and Injection Practices

Now I'm going to ask you some questions about your experiences with syringe access.

C1. **Where did you get most of the syringes you used for injecting drugs in the last 3 months?** (Choose one)

Needle exchange program	01
Needle distribution program or outreach worker	02
From someone who goes to the needle exchange	03
Bought from a pharmacy	04
Bought or got from an unauthorized sources (street, a friend)	05
My own prescription for syringes	06
Shooting gallery or drug dealer	07
Other	08
Don't Know	77
Refuse to Answer	88

If C1 is not equal to 8, then skip to C3.

C2. **Please specify the other place where you obtained most of your needles in the last <u>three months</u>.**

|__|__|__|__|__|__|__|__|__|__|__|__|__|__|__|__|__|__|__|__|__|__|__|__|__|__|__|__|__|__|__|__|__|__|__|__|__|__|__|__|

C3. **In the last 3 months, how many new needles have you obtained for your own use?**

— — — — —	
99997	Don't Know
99998	Refuse to Answer
99999	Not Applicable

C4. **On average, when you get a brand new, never-used needle, how many times do you inject with it before you stop using it or get rid of it?**

	|__|__|__|
Don't Know	777
Refuse to Answer	888

The next several questions are about behaviors related to injection drug use.

C5. **In the last <u>3 months</u>, how many <u>times</u> did you give or loan syringes/needles that you had used to someone else (including a close friend or lover) who then used them?**

Times	|__|__|__|__|
Don't Know	7777
Refuse to Answer	8888

C6. **In the last <u>3 months</u>, how many <u>times</u> did you inject using syringes/needles that <u>you know</u> had been used by someone else (including a close friend or lover)?**

Times	|__|__|__|__|
Don't Know	77777
Refuse to Answer	88888
Not Applicable	99999

C7. **In the last 3 months, have you shared a cooker, spoon, or shaker that someone else used before you?**

Yes	1
No	0
Don't Know	7
Refuse to Answer	8

C8. **In the last 3 months, have you used rinse or mix water that someone else used before you?**

Yes	1
No	0
Don't Know	7
Refuse to Answer	8

C9. **In the last 3 months, have you used a filter or cotton that someone else used before you?**

Yes	1
No	0
Don't Know	7
Refuse to Answer	8

If C9 is equal to 0, then skip to C11.

C10. **In the last 3 months, how frequently have you used a cotton or filter that had been used by someone else?** (Choose one)

0	Less than once a month
1	1 to 4 times a month

2	2 to 6 times a week
3	Once a day
4	2 or more times a day
7	Don't Know
8	Refuse to Answer
9	Not Applicable

C11. **In the last 3 months, have you re-used your own cotton or filter? That is, saved it and then re-wetted it to get residual drug from it or just used it again because an unused cotton or filter was unavailable.**

1	Yes
0	No
7	Don't Know
8	Refuse to Answer
9	Not Applicable

If C11 is equal to 0, then skip to C13.

C12. **In the last 3 months, how frequently have you re-used a cotton or filter?** (Choose one)

0	Less than once a month
1	1 to 4 times a month
2	2 to 6 times a week
3	Once a day
4	2 or more times a day
7	Don't Know
8	Refuse to Answer
9	Not Applicable

C13. **In the last 3 months, have you licked the needle or wetted or moisten a needle with saliva/spit prior to injecting with a needle?**

1	Yes
0	No
7	Don't Know
8	Refuse to Answer
9	Not Applicable

If C13 is equal to 0, then skip to C15.

C14. **In the last 3 months, how often did you wet or moisten a needle with saliva/spit before injecting yourself?** (Choose one)

0	None
1	Some
2	About half
3	Most
4	All
7	Don't Know
8	Refuse to Answer
9	Not Applicable

C15. **In the last 3 months, have you used your saliva/spit to wipe or "clean" a spot on your body that you then injected into?** (Did you clean a place to inject on your body with saliva or spit prior to injecting into

that spot?)

1	Yes
0	No
7	Don't Know
8	Refuse to Answer
9	Not Applicable

If C15 is equal to 0, then skip to C17.

C16. **In the last 3 months, how often did you wipe your injection site with saliva/spit prior to injecting?** (Choose one)

0	None
1	Some
2	About half
3	Most
4	All
7	Don't Know
8	Refuse to Answer
9	Not Applicable

C17. **In the last 3 months, did you inject another person? (Count only injection of illicit substances or misuse of licit substances**)

Yes	1
No	0
Don't Know	7
Refuse to Answer	8
Not Applicable	9

C18. **In the last 3 months, have you been injected by another person? (Count only injection of illicit substances or misuse of licit substances.**
)

1	Yes
0	No
7	Don't Know
8	Refuse to Answer
9	Not Applicable

C19. **In the last 3 months, how often do you inject in public places (e.g., a park, alley, parking lot) or other place where other people can see you inject? (Injecting alone in your tent would not be considered public injection.**) (Choose one)

0	Never (0% of the time)
1	Occasionally (less than 25% of the time)
2	Sometimes (25% to 74% of the time)
3	Usually (75% to 99% of the time)
4	Always (100% of the time)
7	Don't Know
8	Refuse to Answer
9	Not Applicable

C20. **In the last 3 months, how many times have you rushed your injection because you were concerned someone would see or interrupt you?**

____ ____ ____ ____

9997	Don't Know
9998	Refuse to Answer
9999	Not Applicable

C21. **In the last 3 months, in which of the following places have you injected?** (Check all that apply)

__	Your own house/apartment
__	A friend house/apartment
__	An abandoned building
__	A car or other vehicle
__	A shooting gallery
__	A hotel or motel room
__	In public - street, park or alley
__	In a tent
__	In a public restroom
__	In a safe injection facility, safe consumption site
__	In a refresh center (restroom & showers)
__	In a homeless shelter
__	Don't Know
__	Refuse to Answer
__	Not Applicable

C22. **In the last 3 months, where have you usually been located when you injected?** (Choose one)

00	Your own house/apartment
01	A friend's house/apartment
02	An abandoned building
03	A car or other vehicle
04	A shooting gallery
05	A hotel or motel room
06	In public - street, park or alley
07	In a tent
08	In a public restroom
09	In a safe injection facility/safe consumption site
10	In a refresh center (restroom & showers)
11	In a homeless shelter
97	Don't Know
98	Refuse to Answer
99	Not Applicable

For purposes of the next questions, we have defined supervised consumption site as a place where people come to use their own drugs under the supervision of trained workers. People can use in this place, have access to sterile equipment (syringes, cotton, cookers) and receive referrals to health or social services.

C23. **Have you ever used drugs in a supervised consumption site?** 1 Yes

0	No
7	Don't Know
8	Refuse to Answer

9	Not Applicable

If C23 is equal to 0, then skip to instruction before D1.

C24. **In what year did you first use a supervised consumption site (or safe injection or drug use facility)?**

— — — —	yyyy
2097	Don't Know (Year)
2098	Refuse to Answer (Year)
2099	Not Applicable (Year)

C25. **In what city was this supervised consumption room located?**

— —

C26. **In the last 3 months, have you used drugs in a supervised consumption site?**

1	Yes
0	No
7	Don't Know
8	Refuse to Answer
9	Not Applicable

If C26 is equal to 0, then skip to instruction before D1.

C27. **In the last 3 months, how many times have you used drugs at a supervised consumption site?**

— — —	
997	Don't Know
998	Refuse to Answer
999	Not Applicable

If C27 is equal to 0, then skip to instruction before D1.

C28. **In the last 3 months, how many times have you injected drugs at a supervised consumption site?**

— —	
97	Don't Know
98	Refuse to Answer
99	Not Applicable

C29. **In the last 3 months, how many times have you smoked drugs at a supervised consumption site?**

— —	
97	Don't Know
98	Refuse to Answer
99	Not Applicable

C30. **In the last 3 months, how many times have you snorted or swallowed drugs at a supervised consumption site?**

— —	
97	Don't Know
98	Refuse to Answer
99	Not Applicable

D. OVERDOSE

The following items are about overdoses you have experienced or witnessed.

D1. **In the last 3 months, have you overdosed?**

1	Yes
0	No
7	Don't Know
8	Refuse to Answer
9	Not Applicable

If D1 is equal to 0, then skip to D6.

D2. **In the last 3 months, how many times did you overdose?** __ __

97	Don't Know
98	Refuse to Answer
99	Not Applicable

D3. **What drugs had you been using prior to your last overdose(s)? (Include substances and alcohol that you had used in the prior 4 hours before the overdose)** (Check all that apply)

- __ Crack cocaine
- __ Powder cocaine
- __ Heroin
- __ Fentanyl
- __ Prescription opioids
- __ Methamphetamine
- __ Speedball
- __ Goofball
- __ Prescription stimulant
- __ Prescription tranquilizer/Benzodiazepines
- __ Cannabis
- __ Methadone
- __ Buprenorphine
- __ Alcohol
- __ Prescription sedative/barbiturate
- __ Other drug
- __ Don't Know
- __ Refuse to Answer
- __ Not Applicable

If D3P is equal to 0, then skip to D5.

D4. **What was this other drug that you used prior to overdosing in the last 3 months?**

____

D5. **When you overdosed in the last 3 months, did any of the following occur in response to your overdose?** (Check all that apply)

- __ Received naloxone
- __ 911 was called
- __ Transported to ER
- __ Received rescue breathing/CPR
- __ Was examined by EMS

__	Was offered MOUD TX
__	Contact with police
__	Arrested
__	Don't Know
__	Refuse to Answer
__	Not Applicable

D6. **In the last 3 months, have you witnessed a heroin, fentanyl, or prescription opioid overdose?**

1	Yes
0	No
7	Don't Know
8	Refuse to Answer
9	Not Applicable

If D6 is equal to 0, then skip to instruction before E1.

D7. **In the last 3 months, how many heroin or opioid overdoses have you witnessed?**

— — — —	Overdoses
9997	Don't Know
9998	Refuse to Answer
9999	Not Applicable

D8. **In the last 3 months, how many of these witnessed overdoses did you or someone else who was not an EMT or police officer use naloxone or narcan to reverse the overdose?**

— — — —	Times
9997	Don't Know
9998	Refuse to Answer
9999	Not Applicable

If D8 is equal to 0, then skip to D10.

D9. **Of the overdoses you witnessed where naloxone was used, did any of them require more than one dose to revive the person to the best of your knowledge?**

1	Yes
0	No
7	Don't Know
8	Refuse to Answer
9	Not Applicable

D10. **In the last 3 months, how many of these witnessed overdoses did you or someone else call 911?**

— — — —	
9997	Don't Know
9998	Refuse to Answer
9999	Not Applicable

D11. **In the last 3 months, how many of these witnessed overdoses resulted in the person who overdosed being transported to the emergency room?**

— — — —	
9997	Don't Know
9998	Refuse to Answer
9999	Not Applicable

E. WITHDRAWAL

The following questions are about withdrawal symptoms related to substance use.

E1. **In the last 3 months, have you experienced restlessness, bone or muscle aches, runny nose, sweating, cold or hot flashes, anxiety, teary eyes, stomach cramps, nausea, diarrhea, vomiting or other symptoms due to withdrawal from heroin or opiates?** (Also, referred to as being "dopesick.")

1	Yes
0	No
7	Don't Know
8	Refuse to Answer
9	Not Applicable

If E1 is equal to 0, then skip to instruction before E4.

E2. **In the last 3 months, how many times have you experienced heroin or opiate withdrawal or withdrawal symptoms?**

— — — —	Times
9997	Don't Know
9998	Refuse to Answer
9999	Not Applicable

E3. **In the last 3 months, when you were in heroin or opiate withdrawal, how painful were your symptoms?** (Choose one)

0	Not painful
1	Somewhat painful
2	Moderately painful
3	Very painful
4	Extremely painful
7	Don't Know
8	Refuse to Answer
9	Not Applicable

If METSUM is equal to 0, then skip to instruction before E7.

E4. **In the last 3 months, have you experienced trouble sleeping, concentrating, being tired, irritable, agitated, anxious, sad, depressed, or unable to do things you normally do because of withdrawal from amphetamines, methamphetamines, or speed?**

1	Yes
0	No
7	Don't Know
8	Refuse to Answer
9	Not Applicable

If E4 is equal to 0, then skip to instruction before E7.

E5. **In the last 3 months, how many times have you experienced withdrawal or withdrawal symptoms due to amphetamine, methamphetamine or speed?**

— — — —	Times
9997	Don't Know
9998	Refuse to Answer

9999	Not Applicable

E6. **In the last 3 months, when you were in amphetamine, methamphetamine or speed withdrawal, how painful were your symptoms?** (Choose one)

0	Not painful
1	Somewhat painful
2	Moderately painful
3	Very painful
4	Extremely painful
7	Don't Know
8	Refuse to Answer
9	Not Applicable

If COKSUM is equal to 0, then skip to instruction before F1.

E7. **In the last 3 months, have you experienced trouble sleeping or sleeping too much, not being able to concentrate, being irritable, tense, sad, unable to enjoy things you normally do or eating too little or too much due to withdrawal from cocaine or crack cocaine?**

1	Yes
0	No
7	Don't Know
8	Refuse to Answer
9	Not Applicable

If E7 is equal to 0, then skip to instruction before F1.

E8. **In the last 3 months, how many times have you experienced withdrawal or withdrawal symptoms due to cocaine or crack cocaine?**

___ ___ ___ ___	Times
9997	Don't Know
9998	Refuse to Answer
9999	Not Applicable

E9. **In the last 3 months, when you were in powdered cocaine or crack cocaine withdrawal, how painful were your symptoms?** (Choose one)

0	Not painful
1	Somewhat painful
2	Moderately painful
3	Very painful
4	Extremely painful
7	Don't Know
8	Refuse to Answer
9	Not Applicable

F. SEXUAL BEHAVIOR

Now I would like to ask you some personal questions about your sexual behavior. We realize that this is a very personal subject, but your answers are very important to our research. Your answers will remain completely confidential, and I remind you that your name isn't written anywhere on this questionnaire. You may refuse to answer any question you wish, but it is important that you be as honest as you can.

In the following questions, by "having sex" we mean having vaginal or anal sex (or insertive/ receptive sex). We are not interested in sexual activities that involved only oral sex, petting, mutual masturbation, or using sex toys such as dildos.

F1. **Have you had any sex partners in the last 3 months?**

Yes	1
No	0
Don't Know	7
Refuse to Answer	8
Not Applicable	9

If F1 is equal to 0, then skip to F18.

F2. **How many sex partners did you have in the last 3 months?** __ __ __ __

9997	Don't Know
9998	Refuse to Answer
9999	Not Applicable

F3. **In the last 3 months, did you have a steady sex partner?**

Yes	1
No	0
Don't Know	7
Refuse to Answer	8
Not Applicable	9

If F3 is equal to 0, then skip to F8.

F4. **In the last 3 months, when you had sex with your steady sex partner(s), how often were you under the influence of drugs or alcohol?** (Choose one)

0	Never (0% of the time)
1	Occasionally (less than 25% of the time)
2	Sometimes (25% to 74% of the time)
3	Usually (75% to 99% of the time)
4	Always (100% of the time)
7	Don't Know
8	Refuse to Answer
9	Not Applicable

F5. **How frequently did you use a condom when having vaginal or anal sex with your steady sex partner?** (Choose one)

Never (0% of the time)	0
Occasionally (less than 25% of the time)	1
Sometimes (25% to 74% of the time)	2
Usually (75% to 99% of the time)	3
Always (100% of the time)	4
Don't Know	7
Refuse to Answer	8
Not Applicable	9

F6. **Does your steady sex partner use drugs?** 1

Yes

0 No

7 Don't Know

8 Refuse to Answer

9 Not Applicable

If F6 is equal to 0, then skip to F8.

F7. **Does your steady sexual partner inject drugs?**

Yes	1
No	0
Don't Know	7
Refuse to Answer	8
Not Applicable	9

F8. **In the last <u>3 months</u>, did you have any sex partners who paid you in cash or drugs for sex?**

Yes	1
No	0
Don't Know	7
Refuse to Answer	8
Not Applicable	9

If F8 is equal to 0, then skip to F13.

F9. **In the last 3 months, when you had sex with your paying sex partner(s), how often were you under the influence of drugs or alcohol?** (Choose one)

0	Never (0% of the time)
1	Occasionally (less than 25% of the time)
2	Sometimes (25% to 74% of the time)
3	Usually (75% to 99% of the time)
4	Always (100% of the time)
7	Don't Know
8	Refuse to Answer
9	Not Applicable

F10. **How frequently did you use condoms when having vaginal or anal sex with your paying sex partners?** (Choose one)

Never (0% of the time)	0
Occasionally (less than 25% of the time)	1
Sometimes (25% to 74% of the time)	2
Usually (75% to 99% of the time)	3
Always (100% of the time)	4
Don't Know	7
Refuse to Answer	8
Not Applicable	9

F11. **Did any of your paying sex partners use drugs?** 1

Yes

0 No

7 Don't Know

8 Refuse to Answer

9	Not Applicable

If F11 is equal to 0, then skip to F13.

F12. **Did any of your paying sex partner(s) inject drugs?**

Yes	1
No	0
Don't Know	7
Refuse to Answer	8
Not Applicable	9

F13. **In the last 3 months, did you have a casual sex partner (a sex partner who was not steady and not paid)?**

Yes	1
No	0
Don't Know	7
Refuse to Answer	8
Not Applicable	9

If F13 is equal to 0, then skip to F18.

F14. **In the last 3 months, when you had sex with your casual sex partner(s), how often were you under the influence of drugs or alcohol?** (Choose one)

0	Never (0% of the time)
1	Occasionally (less than 25% of the time)
2	Sometimes (25% to 74% of the time)
3	Usually (75% to 99% of the time)
4	Always (100% of the time)
7	Don't Know
8	Refuse to Answer
9	Not Applicable

F15. **How frequently did you use a condom when having vaginal or anal sex with your casual sex partner(s)?** (Choose one)

Never (0% of the time)	0
Occasionally (less than 25% of the time)	1
Sometimes (25% to 74% of the time)	2
Usually (75% to 99% of the time)	3
Always (100% of the time)	4
Don't Know	7
Refuse to Answer	8
Not Applicable	9

F16. **Did any of your casual sex partners use drugs?** 1
Yes

0	No
7	Don't Know
8	Refuse to Answer
9	Not Applicable

If F16 is equal to 0, then skip to F18.

F17. **Did any of your casual sexual partner(s) inject drugs?**

Yes	1
No	0
Don't Know	7
Refuse to Answer	8
Not Applicable	9

F18. **What do you consider your sexual orientation to be?**
(Read list aloud.) (Choose one)

Heterosexual	1
Gay or lesbian	2
Bisexual	3
Asexual - nonsexual	4
Don't Know	7
Refuse to Answer	8
Not Applicable	9

G. HEALTH

The next set of questions are about your health, use of preventive services, and attitudes toward treatments,

G19. **Have you ever received a vaccination to protect you from Hepatitis A virus?**

1	Yes
0	No
7	Don't Know
8	Refuse to Answer
9	Not Applicable

G20. **Has a doctor, nurse, or health provider ever told you that you were infected with hepatitis A virus?**

1	Yes
0	No
7	Don't Know
8	Refuse to Answer
9	Not Applicable

G21. **Has a doctor, nurse, or counselor ever told you that you are HIV positive?**

Yes	1
No	0
Don't Know	7
Refuse to Answer	8

If G21 is equal to 0, then skip to instruction before G23.

G22. **Are you currently receiving treatment for HIV by taking ART medications and visiting your physician once every 6 months?**

1	Yes	
0	No	***Skip to instruction before G24***
7	Don't Know	

8 Refuse to Answer

9 Not Applicable ***Skip to instruction before G24***

If G21 is equal to 1, then skip to G34.

If G22 is equal to 0, then skip to G34.

G23. **Have you ever heard of a pill that is safe and effective at lowering transmission of HIV if taken daily prior to exposure to HIV (also known as PrEP or pre-exposure prophylaxis)?**

1 Yes

0 No

7 Don't Know

8 Refuse to Answer

9 Not Applicable

If G23 is equal to 0, then skip to G25.

G24. **Are you currently taking a daily pill to lower your risk for HIV transmission? This pill is typically referred to as PrEP or pre-exposure prophylaxis.**

1 Yes

0 No

7 Don't Know

8 Refuse to Answer

9 Not Applicable

If G24 is equal to 1, then skip to G26.

G25. **Would you be willing to take a once a day pill, everyday to lower your risk of becoming HIV infected by 90%?**

1 Yes

0 No

7 Don't Know

8 Refuse to Answer

9 Not Applicable

G26. **Have you ever tried to get PrEP?** 1 Yes

0 No

7 Don't Know

8 Refuse to Answer

9 Not Applicable

If G26 is equal to 0, then skip to G31.

G27. **Did you ever receive a prescription for PrEP?** 1 Yes

0 No

7 Don't Know

8 Refuse to Answer

9 Not Applicable

If G27 is equal to 0, then skip to G29.

G28. **Were you able to fill any PrEP prescription that you have received?** 1

Yes

0 No

7 Don't Know

8 Refuse to Answer

9 Not Applicable

If G28 is equal to 0, then skip to G30.

G29. **Did you ever take the PrEP prescribed to you?** 1 Yes

0 No

7 Don't Know

8 Refuse to Answer

9 Not Applicable

G30. **Have you ever taken PrEP not prescribed to you?** 1 Yes

0 No

7 Don't Know

8 Refuse to Answer

9 Not Applicable

G31. **Have you ever heard of a pill that can be taken for 30 days after a single high-risk event to stop HIV seroconversion (also call PEP or post-exposure prophylaxis)?**

1 Yes

0 No

7 Don't Know

8 Refuse to Answer

9 Not Applicable

If G31 is equal to 0, then skip to G33.

G32. **In the last 3 months, have you ever taken an antiretroviral pill after a single high-risk event to stop HIV seroconversion?**

1 Yes

0 No

7 Don't Know

8 Refuse to Answer

9 Not Applicable

If G32 is equal to 1, then skip to G34.

G33. **Would you be willing to take a once a day pill for 30 days, after a single high-risk event to prevent HIV seroconversion?**

1 Yes

0 No

7 Don't Know

8 Refuse to Answer

9 Not Applicable

G34. **Have you ever tested positive for hepatitis C virus or HCV infection or been told by a health care professional that you are infected with the hepatitis C virus or HCV?**

1 Yes

0	No
7	Don't Know
8	Refuse to Answer
9	Not Applicable

If G34 is equal to 0, then skip to G36.

G35. **Have you ever or are you currently receiving treatment for hepatitis C virus or HCV infection?**

1	Yes
0	No
7	Don't Know
8	Refuse to Answer
9	Not Applicable

G36. **Have you ever been diagnosed with mental health disorder or a psychiatric illness (e.g. major depression, bipolar disorder, anxiety, PTSD, schizophrenia)?**

1	Yes
0	No
7	Don't Know
8	Refuse to Answer
9	Not Applicable

If G36 is equal to 0, then skip to G42.

G37. **Which of the following mental health diagnoses have you received? (Read list)** (Check all that apply)

__ Depression
__ Bipolar disorder/manic depression
__ Schizophrenia
__ PTSD or Post traumatic stress disorder
__ Anxiety
__ Other psychiatric illness
__ Don't Know
__ Refuse to Answer
__ Not Applicable

If G37F is equal to 0, then skip to G39.

G38. **What was this other mental health disorder or psychiatric illness?**

____
____
____

G39. **Are you currently taking any doctor prescribed medications for any of your mental health diagnoses?**

1	Yes
0	No
7	Don't Know
8	Refuse to Answer
9	Not Applicable

If G39 is equal to 0, then skip to G42.

G40. **For which diagnoses are you prescribed medications?** (Check all that apply)

__	Depression
__	Bipolar disorder
__	Schizophrenia
__	PTSD
__	Anxiety
__	Other
__	Don't Know
__	Refuse to Answer
__	Not Applicable

If G40F is equal to 0, then skip to G42.

G41. **What is the other diagnosis that you have been prescribed medications for?**

———

G42. **In the last 3 months, have you been detained under a 5150 order or involuntarily hospitalized due to a mental health problem?**

1	Yes
0	No
7	Don't Know
8	Refuse to Answer
9	Not Applicable

If G42 is equal to 0, then skip to G44.

G43. **How many times were you detained in the last 3 months?** __ __

97	Don't Know
98	Refuse to Answer
99	Not Applicable

G44. **In the last 3 months, did you voluntarily hospitalize yourself due to a mental health disorder?**

1	Yes
0	No
7	Don't Know
8	Refuse to Answer
9	Not Applicable

G45. **In the last 3 months, have you had an abscess related to injection drug use, even if it did not "come to a head," drain, or require treatment of any kind?**

1	Yes
0	No
7	Don't Know
8	Refuse to Answer
9	Not Applicable

If G45 is equal to 0, then skip to G47.

G46. **How many abscesses have you had in the last 3 months?** (It is fine to estimate.)

__ __ __ __

9997	Don't Know
9998	Refuse to Answer
9999	Not Applicable

G47. **In the past 3 months, have you experienced high fevers, shaking chills, night sweats or other symptoms due to a potentially untreated infection?**

1	Yes
0	No
7	Don't Know
8	Refuse to Answer
9	Not Applicable

If G47 is equal to 0, then skip to G49.

G48. **Were those symptoms accompanied by increased shortness of breath, weight loss, back pain, chest pain, bloody urine, painful spots on your fingers or toes, painless red spots on your fingers or toes, swelling in your legs, loss of or changes in vision, or new headaches?**

1	Yes
0	No
7	Don't Know
8	Refuse to Answer
9	Not Applicable

G49. **Have you ever been diagnosed with infective endocarditis (an infection to your heart valve)?**

1	Yes
0	No
7	Don't Know
8	Refuse to Answer
9	Not Applicable

If G49 is equal to 0, then skip to G54.

G50. **Did you complete the full treatment course prescribed to you? Usually, this includes 4-8 weeks of antibiotics and sometimes surgery to replace a heart valve.**

1	Yes
0	No
7	Don't Know
8	Refuse to Answer
9	Not Applicable

If G50 is equal to 0, then skip to instruction before G52.

G51. **Why did you not complete the full treatment course?** (Check all that apply)

__	Could not pay for treatment
__	Did not feel treatment was appropriate
__	Did not receive treatment for withdrawal symptoms
__	Did not think treatment was necessary
__	Was not treated fairly by medical staff
__	Was kicked out of care for any reason
__	Was kicked out of care for drug use

__	Other
__	Don't Know
__	Refuse to Answer
__	Not Applicable

If G51H is equal to 0, then skip to G53.

G52. **For what other reasons did you not complete treatment?**

____

G53. **In the last 3 months, has a doctor, nurse, or medical provider told you that you have infective endocarditis (an infection in your heart valve)?**

1	Yes
0	No
7	Don't Know
8	Refuse to Answer
9	Not Applicable

G54. **Has a health care professional ever told you that you have [Diabetes, High blood pressure, ...]?**

Yes	1
No	0
Don't Know	7
Refuse to Answer	8
Not Applicable	9

	Diabetes	High blood pressure/ hypertension	High cholesterol	Congestive Heart Failure/ Heart Attack	Stroke	MRSA
G54	|__|	|__|	|__|	|__|	|__|	|__|

G55. **In the last 3 months, how many times have you been seen in an emergency room for a health problem?**

___	
97	Don't Know
98	Refuse to Answer
99	Not Applicable

G56. **How many times have you been hospitalized overnight for any health issue in the last 3 months?**

____	Times
9997	Don't Know
9998	Refuse to Answer
9999	Not Applicable

If G56 is equal to 0, then skip to instruction before G58.

G57. **How many nights did you spend in the hospital in the last 3 months?**

___	
997	Don't Know

998	Refuse to Answer
999	Not Applicable

If A3 is equal to 0, then skip to instruction before G59&C.

If A3 is greater than 1, then skip to instruction before G59&C.

G58. **In the last 3 months, have you experienced any of the following gynecological symptoms or conditions? (Read list - selected not applicable if none are mentioned)** (Check all that apply)

__ Need for a pregnancy test
__ Concern about a pregnancy
__ Miscarriage
__ Missed a period for a month or more
__ Need for an abortion
__ An abortion
__ Don't Know
__ Refuse to Answer
__ Not Applicable

The next items about your experiences during the first 12 months of the COVID-19 pandemic.

G59. **At any point during [Apr to June 2020, Jul to Sep 2020, ...] did you receive a COVID-19 test?**

Yes	1
No	0
Don't Know	7
Refuse to Answer	8
Not Applicable	9

G60. **At any point during [Apr to June 2020, Jul to Sep 2020, ...] did you test positive for COVID-19?**

Yes	1
No	0
Don't Know	7
Refuse to Answer	8
Not Applicable	9

G61. **At any point during [Apr to June 2020, Jul to Sep 2020, ...] did you have to quarantine because of potential exposure to someone who was COVID-19?**

Yes	1
No	0
Don't Know	7
Refuse to Answer	8
Not Applicable	9

G62. **At any point during [Apr to June 2020, Jul to Sep 2020, ...] were you incarcerated in jail or prison?**

Yes	1
No	0
Don't Know	7
Refuse to Answer	8
Not Applicable	9

G63. **At any point during [Apr to June 2020, Jul to Sep 2020, ...] were you housed to prevent exposure to COVID-19? Probe: Housed in project roomkey or other temporary housing to help you shelter-in-place?**

Yes	1
No	0
Don't Know	7
Refuse to Answer	8
Not Applicable	9

	Apr to June 2020	Jul to Sep 2020	Oct to Dec 2020	Jan to Mar 2021
G59	|__|	|__|	|__|	|__|
G60	|__|	|__|	|__|	|__|
G61	|__|	|__|	|__|	|__|
G62	|__|	|__|	|__|	|__|
G63	|__|	|__|	|__|	|__|

G64. **Have you received a vaccination for COVID-19?** 1
Yes

0 No
7 Don't Know
8 Refuse to Answer
9 Not Applicable

If G64 is equal to 0, then skip to G66.

G65. **What month and year did you receive the COVID-19 vaccine?**

— — — — — — — — — — — — — — — — — — — —

G66. **Do you have any of the following types of health benefits? (Read List)** (Check all that apply)

Private insurance	|__|
Medicaid or Medi-Cal	|__|
Medicare	|__|
Veterans benefits	|__|
Covered California	|__|
Other	|__|
No insurance	|__|
Don't Know	|__|
Refuse to Answer	|__|
Not Applicable	|__|

If G66F is equal to 0, then skip to instruction before G68.

G67. **What is this other kind of health insurance?**

— —

The next half dozen questions are about chronic pain.

G68. **In the last 3 months, have you experienced any chronic or ongoing pain due to an injury or other ailment (like arthritis or migraine headaches, but not counting withdrawal symptoms)?**

1	Yes
0	No
7	Don't Know
8	Refuse to Answer
9	Not Applicable

If G68 is equal to 0, then skip to instruction before G76.

G69. **How would you rate your chronic physical pain on a 0-9 scale at the present time or right now, where 0 is 'no pain' and 9 is 'pain as bad as it could be?'**

00	No pain
01	
02	
03	
04	
05	
06	
07	
08	
09	Worst pain
97	Don't Know
98	Refuse to Answer
99	Not Applicable

G70. **In the past 3 months, how intense was your worst chronic physical pain rated on a 0-9 scale, where 0 is 'no pain' and 9 is 'pain as bad as it could be?'**

00	No pain
01	
02	
03	
04	
05	
06	
07	
08	
09	Worst pain
97	Don't Know
98	Refuse to Answer
99	Not Applicable

G71. **In the past 3 months, on average, how intense was your chronic physical pain rated on a 0-9 scale? (That is your usual pain at times you were experiencing pain.)**

00	No pain
01	
02	
03	
04	
05	
06	

07	
08	
09	Worst pain
97	Don't Know
98	Refuse to Answer
99	Not Applicable

G72. **About how many <u>DAYS</u> in the last 3 months have you been kept from your usual activities (work, school, housework) because of chronic physical pain?**

— — —	DAYS
997	Don't Know
998	Refuse to Answer
999	Not Applicable

G73. **In the past 3 months, how much has this chronic physical pain interfered with your daily activities on a 0-9 scale where 0 is 'no interference' and 9 is 'extreme change'?**

00	No interference
01	
02	
03	
04	
05	
06	
07	
08	
09	Extreme change
997	Don't Know
998	Refuse to Answer
999	Not Applicable

G74. **In the past 3 months, how much has chronic physical pain changed your ability to take part in recreational, social, and family activities where 0 is 'no change' and 9 is 'extreme change'?**

00	No change
01	
02	
03	
04	
05	
06	
07	
08	
09	Extreme change
997	Don't Know
998	Refuse to Answer
999	Not Applicable

G75. **In the past 3 months, how has this chronic physical pain changed your ability to work (including housework) where 0 is 'no change' and 9 is 'extreme change'?**

00	No change

01	
02	
03	
04	
05	
06	
07	
08	
09	Extreme change
997	Don't Know
998	Refuse to Answer
999	Not Applicable

The next set of questions are about how people have treated you and people like you in the last 3 months.

G76. **In the last 3 months, how often have people spoken or acted towards you in a way that felt like they were attacking your dignity or demeaning you?** (Choose one)

1	Never
2	1 to 2 times
3	3-10 times
4	About once a month
5	About once or twice a week
6	Daily or more
7	Don't Know
8	Refuse to Answer
9	Not Applicable

If G76 is equal to 1, then skip to G79.

G77. **What aspect of you or your life did you think they were demeaning you about?** (Check all that apply)

__	My drug use
__	The way I use drugs (injection)
__	My personality
__	The way I look
__	My clothing
__	Not having a job
__	Lack of education
__	People I hung out with
__	People I have sex with
__	My race
__	My gender
__	My sexual orientation
__	My weight
__	My personal hygiene (cleanliness)
__	How I earn money
__	Being poor
__	My HIV or HCV status

__ Having children with different partners
__ My housing situation
__ HIV status
__ HCV status
__ Don't Know
__ Refuse to Answer
__ Not Applicable

G78. **Please tell me who are the people and/or groups who have attacked your dignity or demeaned you in the way that hurt you the most?** (Check all that apply)

__ MOTHER
__ FATHER
__ SIBLING
__ EX-SPOUSE/LOVER
__ OTHER RELATIVES
__ FRIENDS
__ NEIGHBORS
__ BOSS/EMPLOYER
__ CO-WORKERS
__ DRUG DEALER
__ COUNSELOR/SOCIAL WORKER
__ PROBATION/PAROLE OFFICER
__ POLICE OFFICER
__ STOREOWNER
__ STRANGER
__ HOSPITAL OR CLINIC STAFF/DOCTOR
__ DRUG TREATMENT STAFF
__ SECURITY GUARD
__ PHARMACY STAFF
__ SEP/SSP STAFF
__ Don't Know
__ Refuse to Answer
__ Not Applicable

G79. **In the last 3 months, how often have you been present when someone spoke or acted towards someone else in a way that attacked their dignity or demeaned them?** (Choose one)

1 Never
2 1 to 2 times
3 3-10 times
4 About once a month
5 About once or twice a week
6 Daily or more
7 Don't Know
8 Refuse to Answer
9 Not Applicable

If G79 is equal to 1, then skip to instruction before H1.

G80. **What aspect of their life did you think they were being demeaned about?** (Check all that apply)

__ Their drug use
__ The way they use drugs (injection)
__ Their personality
__ The way they look
__ Their clothing
__ Not having a job
__ Lack of education
__ People they hung out with
__ People they have sex with
__ Their race
__ Their gender
__ Their sexual orientation
__ Their weight
__ Their personal hygiene (cleanliness)
__ How they earn money
__ Being poor
__ Their HIV or HCV status
__ Having children with different partners
__ Their housing situation
__ Their HIV status
__ Their HCV status
__ Don't Know
__ Refuse to Answer
__ Not Applicable

G81. **Please tell me who are the people and/or groups who have attacked this other person's dignity or demeaned them in the way that hurt them the most?** (Check all that apply)

__ MOTHER
__ FATHER
__ SIBLING
__ EX-SPOUSE/LOVER
__ OTHER RELATIVES
__ FRIENDS
__ NEIGHBORS
__ BOSS/EMPLOYER
__ CO-WORKERS
__ DRUG DEALER
__ COUNSELOR/SOCIAL WORKER
__ PROBATION/PAROLE OFFICER
__ POLICE OFFICER
__ STOREOWNER
__ STRANGER
__ HOSPITAL OR CLINIC STAFF/DOCTOR
__ DRUG TREATMENT STAFF
__ SECURITY GUARD
__ PHARMACY STAFF
__ SEP/SSP STAFF

__	Don't Know
__	Refuse to Answer
__	Not Applicable

H. VIOLENCE

The next questions are about threats and violence that may have happened to you in the past 3 months.

H1. **In the past 3 months, has anybody threatened you with a knife, gun, or other weapon?**

1	Yes
0	No
7	Don't Know
8	Refuse to Answer
9	Not Applicable

H2. **In the past 3 months, has anybody punched, slapped, kicked, or physically hurt you?**

1	Yes
0	No
7	Don't Know
8	Refuse to Answer
9	Not Applicable

H3. **In the past 3 months, has anybody used a knife, gun, club, or other weapon against you?**

1	Yes
0	No
7	Don't Know
8	Refuse to Answer
9	Not Applicable

H4. **In the past 3 months, has somebody used physical force or threats to make you have vaginal, anal, or oral sex with them?**

1	Yes
0	No
7	Don't Know
8	Refuse to Answer
9	Not Applicable

H5. **In the past 3 months, have any of your belongings been stolen?** 1 Yes

0	No
7	Don't Know
8	Refuse to Answer
9	Not Applicable

H6. **In the past 3 months, have you been attacked by a stranger on the street?** 1 Yes

0	No
7	Don't Know
8	Refuse to Answer

9 Not Applicable

I. Law Enforcement

Next I am going to ask you questions about your contacts with security guards, law enforcement, and criminal justice systems.

I1. **In the last 3 months, have you had any contact with private security guards? (do not count police who work for a city, county or state).**

1	Yes
0	No
7	Don't Know
8	Refuse to Answer
9	Not Applicable

If I1 is equal to 0, then skip to I3.

I2. **How many times have you had contact with security guards in the last 3 months?**

— — — —	Times
9997	Don't Know
9998	Refuse to Answer
9999	Not Applicable

I3. **In the past 3 months, have you had contact with the police?** 1
Yes

0	No
7	Don't Know
8	Refuse to Answer
9	Not Applicable

If I3 is equal to 0, then skip to I8.

I4. **How many times have you had contact with the police in the past 3 months**

— — — —	Times
9997	Don't Know
9998	Refuse to Answer
9999	Not Applicable

If I4 is equal to 0, then skip to I8.

I5. **Have you been arrested in the last 3 months?** 1
Yes

0	No
7	Don't Know
8	Refuse to Answer
9	Not Applicable

If I5 is equal to 0, then skip to I8.

I6. **How many times have you been arrested in the last 3 months?** — —
Times

97	Don't Know
98	Refuse to Answer

99	Not Applicable

I7. **How many nights have you spent in jail in the last 3 months?** __ __

97	Don't Know
98	Refuse to Answer
99	Not Applicable

I8. **Have you been on probation at any point in the last 3 months?** 1 Yes

0	No
7	Don't Know
8	Refuse to Answer
9	Not Applicable

I9. **Have you been on parole at any point in the last 3 months?** 1 Yes

0	No
7	Don't Know
8	Refuse to Answer
9	Not Applicable

J. DRUG TREATMENT

The next set of questions are about your experiences with substance use treatment.

J1. **In the last 3 months, have you participated in any treatment program, not counting self-help, NA, CA, or AA?**

1	Yes
0	No
7	Don't Know
8	Refuse to Answer
9	Not Applicable

If J1 is equal to 0, then skip to J5.

J2. **In the last 3 months, have you participated in [Methadone detoxification, Methadone maintenance, ...] ?**

Yes	1
No	0
Refuse to Answer	8
Not Applicable	9

	Methadone detoxification	Methadone maintenance	Buprenorphine detox	Buprenorphine maintenance	Outpatient W/O MAT	Inpatient hospital	Residential W/O MAT	Vivitrol/ naltrexone
J2	|__|	|__|	|__|	|__|	|__|	|__|	|__|	|__|

J3. **Are you currently enrolled in any kind of substance use treatment?** 1

Yes

0	No
7	Don't Know
8	Refuse to Answer
9	Not Applicable

J4. **Which treatments are your currently receiving?** (Check all that apply)

__ Methadone detox
__ Methadone maintenance
__ Buprenorphine
__ Outpatient
__ Inpatient hospital
__ Residential
__ Vivitrol/Naltrexone
__ Don't Know
__ Refuse to Answer
__ Not Applicable

J5. **In the last 3 months, have you tried, but been unable to get into drug treatment?**

1	Yes
0	No
7	Don't Know
8	Refuse to Answer
9	Not Applicable

If J5 is equal to 0, then skip to instruction before J7.

J6. **Which types of treatment did you try to enter in the last 3 months?** (Check all that apply)

__ Methadone detoxification
__ Methadone maintenance
__ Buprenorphine (suboxone or subutex) detox
__ Buprenorphine/suboxone maintenance
__ Outpatient
__ Inpatient hospital
__ Residential
__ Vivitrol/naltrexone
__ Don't Know
__ Refuse to Answer
__ Not Applicable

The following statements refer to heroin, fentanyl, opioids, methamphetamine, cocaine (but not cannabis or alcohol). Please tell me how much you strongly agree, agree, neutral or disagree, or strongly disagree with each statement.

J7. **My drug use is a problem for me.** (Choose one)

0	Strongly disagree
1	Disagree
2	Neutral
3	Agree
4	Strongly agree

		7	Don't Know
		8	Refuse to Answer
		9	Not Applicable
J8.	**My drug use is more trouble than it is worth** (Choose one)	0	Strongly disagree
		1	Disagree
		2	Neutral
		3	Agree
		4	Strongly agree
		7	Don't Know
		8	Refuse to Answer
		9	Not Applicable
J9.	**My drug use is under control.** (Choose one)	0	Strongly disagree
		1	Disagree
		2	Neutral
		3	Agree
		4	Strongly agree
		7	Don't Know
		8	Refuse to Answer
		9	Not Applicable
J10.	**I plan to quit using drugs in the next 6 months.** (Choose one)	0	Strongly disagree
		1	Disagree
		2	Neutral
		3	Agree
		4	Strongly agree
		7	Don't Know
		8	Refuse to Answer
		9	Not Applicable
J11.	**I plan to quit using drugs in the next 30 days.** (Choose one)	0	Strongly disagree
		1	Disagree
		2	Neutral
		3	Agree
		4	Strongly agree
		7	Don't Know
		8	Refuse to Answer
		9	Not Applicable
J12.	**I am ready to quit using drugs right now.** (Choose one)	0	Strongly disagree
		1	Disagree
		2	Neutral
		3	Agree
		4	Strongly agree
		7	Don't Know

8 Refuse to Answer
9 Not Applicable

K. Sleep and subsistence

We are almost done.

K1. **In the last 3 months, how would you rate your sleep quality?** (Choose one) 0 Very bad

1 Fairly bad
2 Fairly good
3 Very good
7 Don't Know
8 Refuse to Answer
9 Not Applicable

K2. **In the last 3 months, how many hours of actual sleep do you usually get per day?**

__ __ HOURS
97 Don't Know (Hours)
98 Refuse to Answer (Hours)
99 Not Applicable (Hours)

K3. **In the last 3 months, has finding shelter been a problem for you usually, sometimes, rarely or never?** (Choose one)

1 Never
2 Rarely
3 Sometimes
4 Usually
7 Don't Know
8 Refuse to Answer
9 Not Applicable

K4. **In the last 3 months, has getting enough to eat been a problem for you usually, sometimes, rarely, or never?** (Choose one)

1 Never
2 Rarely
3 Sometimes
4 Usually
7 Don't Know
8 Refuse to Answer
9 Not Applicable

K5. **In the last 3 months, has obtaining clothes been a problem for you usually, sometimes, rarely, or never?** (Choose one)

1 Never
2 Rarely
3 Sometimes
4 Usually
7 Don't Know
8 Refuse to Answer

9	Not Applicable

K6. **In the last 3 months, has finding a place to wash up been a problem for you usually, sometimes, rarely, or never?** (Choose one)

1	Never
2	Rarely
3	Sometimes
4	Usually
7	Don't Know
8	Refuse to Answer
9	Not Applicable

K7. **In the last 3 months, has finding a place to go to the bathroom been a problem for you usually, sometimes, rarely, or never?** (Choose one)

1	Never
2	Rarely
3	Sometimes
4	Usually
7	Don't Know
8	Refuse to Answer
9	Not Applicable

K8. **FOR INTERVIEWER ONLY; How would you rate the information collected in this interview?** (Choose one)

0	Completely unreliable
1	Not sure if reliable
2	Reliable
7	Don't Know
8	Refuse to Answer
9	Not Applicable

If NUMOPIOID is greater than 0, then skip to end of questionnaire.

This respondent did not report any injection drug use or no opioid use in the last 3 months. They are ineligible for the study. Thank them for their time and end the interview.
